# Discordance of Small Dense LDL Cholesterol Beyond LDL Cholesterol or Non–HDL Cholesterol and Carotid Plaque

**DOI:** 10.1016/j.jacasi.2025.04.015

**Published:** 2025-07-08

**Authors:** Jinqi Wang, Xiaoyu Zhao, Yanchen Zhao, Rui Jin, Yunfei Li, Jiahe Wang, Yueruijing Liu, Zhiyuan Wu, Xiuhua Guo, Lixin Tao

**Affiliations:** a Beijing Key Laboratory of Environment and Aging, Department of Epidemiology and Health Statistics, School of Public Health, Capital Medical University, Beijing, China; b Harvard T.H. Chan School of Public Health, Boston, Massachusetts, USA; c National Institute for Data Science in Health and Medicine, Capital Medical University, Beijing, China; d School of Medical and Health Sciences, Edith Cowan University, Perth, Australia

**Keywords:** carotid plaque, large buoyant low-density lipoprotein cholesterol, residual risks, sdLDL-C/lbLDL-C ratio, sdLDL-C/LDL-C ratio, small dense low-density lipoprotein cholesterol

## Abstract

**Background:**

Different low-density lipoprotein (LDL) particles exhibit distinct proatherogenic properties.

**Objectives:**

This study sought to evaluate associations of small dense low-density lipoprotein cholesterol (sdLDL-C), large buoyant low-density lipoprotein cholesterol (lbLDL-C), sdLDL-C/LDL-C ratio, and sdLDL-C/lbLDL-C ratio with carotid plaque (CP) risk in the general population, and to perform discordance analyses to determine which biomarker better reflects CP risk beyond low-density lipoprotein cholesterol (LDL-C) and non–high-density lipoprotein cholesterol (non-HDL-C).

**Methods:**

This study enrolled 20,369 participants from Beijing Health Management Cohort. Discordant sdLDL-C, lbLDL-C, or ratio metrics (sdLDL-C/LDL-C and sdLDL-C/lbLDL-C) relative to LDL-C or non-HDL-C, and discordant ratio metrics relative to sdLDL-C, were defined by residual differences and median values. Logistic regression models were used to estimate ORs and 95% CIs.

**Results:**

In this study, higher levels of sdLDL-C (OR: 1.354; 95% CI: 1.299-1.410), sdLDL-C/LDL-C ratio (OR: 1.196; 95% CI: 1.148-1.247), and sdLDL-C/lbLDL-C ratio (OR: 1.153; 95% CI: 1.110-1.197) were more strongly associated with increased odds of CP than lbLDL-C (OR: 1.110; 95% CI: 1.070-1.151). Additionally, discordantly high sdLDL-C or low lbLDL-C relative to LDL-C or non-HDL-C were associated with increased odds of CP, whereas discordantly low sdLDL-C or high lbLDL-C were associated with reduced odds. Finally, discordantly high sdLDL-C/LDL-C and sdLDL-C/lbLDL-C ratios relative to LDL-C, non-HDL-C, or sdLDL-C were linked to increased odds of CP.

**Conclusions:**

The sdLDL-C, sdLDL-C/LDL-C, and sdLDL-C/lbLDL-C, but not lbLDL-C, are superior to LDL-C and non-HDL-C in identifying individuals at increased risk of CP. The sdLDL-C/LDL-C and sdLDL-C/lbLDL-C ratios may capture additional risk information beyond sdLDL-C.

Cardiovascular diseases (CVDs) have emerged as a predominant cause of mortality and a pressing global public health concern.^1^ Atherosclerosis is the primary pathological process underlying CVDs, with abnormal lipid metabolism as a substantial contributor.^2^^,^^3^ Reducing low-density lipoprotein cholesterol (LDL-C) levels, primarily through the use of statins, is the leading therapeutic target for primary and secondary prevention of atherosclerosis-related diseases.^4^, ^5^, ^6^, ^7^ However, even with a substantial reduction in LDL-C levels, patients still confront a noteworthy residual risk for CVDs.^8^^,^^9^ Thus, it is urgently necessary to explore new potential biomarkers to combat these residual risks.

Low-density lipoprotein (LDL) particles are heterogeneous in lipid content, size, and density and can be categorized into small dense low-density lipoprotein (sdLDL) and large buoyant low-density lipoprotein (lbLDL).^10^, ^11^, ^12^ These particles exhibit discrepant potential proatherogenic properties. Compared with lbLDL, the particles of sdLDL are smaller in size and have greater ability to penetrate the arterial wall, lower binding affinity for the LDL receptor, longer plasma residence time, and higher susceptibility to oxidation,^13^^,^^14^ and thus may become more atherogenic. Accumulating evidence confirmed that elevated levels of small dense low-density lipoprotein cholesterol (sdLDL-C) were linked with an elevated risk of CVDs.^15^, ^16^, ^17^, ^18^, ^19^ The Copenhagen General Population Study found that higher levels of sdLDL-C, rather than large buoyant low-density lipoprotein cholesterol (lbLDL-C), were related to incident ischemic stroke.^19^ To date, no study has comprehensively evaluated the distinct associations of sdLDL-C and lbLDL-C with carotid plaque risk in the general population.

In addition, there is ongoing debate over whether sdLDL-C can accurately reflect the risk of atherosclerosis beyond established cardiovascular risk factors, including LDL-C and non–HDL-C. One study found that sdLDL-C offers additional CVD risk information relative to non-HDL-C,^20^ whereas another reported no significant association between discordantly elevated sdLDL-C relative to LDL-C and coronary heart disease,^21^ leaving the relationship between discordances in sdLDL-C, lbLDL-C relative to total LDL-C, or non-HDL-C and carotid plaque risk unclear. Notably, considering that different subtypes of LDL-C may have different atherogenic effects, we infer that the ratios of sdLDL-C to total LDL-C (sdLDL-C/LDL-C) and to lbLDL-C (sdLDL-C/lbLDL-C) may convey valuable clinical information.^22^^,^^23^ Whether these two ratio indices could serve as more reliable indicators than other lipoprotein particles in identifying atherosclerosis risk is also an intriguing, unresolved question.

To address this knowledge gap, the present study aimed to: 1) analyze the associations of sdLDL-C, lbLDL-C, sdLDL-C/LDL-C ratio, and sdLDL-C/lbLDL-C ratio with the risk of carotid plaque; and 2) perform discordance analyses to determine which of these indices can more accurately reflect residual carotid plaque risk. We hypothesized that sdLDL-C–related indices would be superior to total LDL-C and non-HDL-C in identifying the atherosclerosis-related risks.

## Methods

### Study design and Population

The Beijing Health Management Cohort study is a large-scale prospective cohort study conducted in Beijing, China, that was previously described in detail.^24^ The purpose of this study is to identify risk factors and biomarkers for multiple chronic conditions, therefore promoting the prevention, treatment, and management of these diseases. Participants were recruited to undergo comprehensive regular physical examinations in the 2 biggest health examination centers in Beijing. The uniform physical examination package included the ultrasonographic examination of the carotid arteries, face-to-face questionnaire survey, physical measurement, and blood test.

We conducted this analysis based on 22,769 underwent health examination in 2020 and 2021 from the Beijing Health Management Cohort study. A total of 39 participants diagnosed with malignant tumor, 848 participants without color Doppler ultrasound examinations of carotid artery and diagnosis information of CVD, and 1,513 participants without examination of sdLDL-C were excluded. Finally, 20,369 participants were enrolled in the study. The flow chart is shown in Supplemental Figure 1.

This study was in accordance with the principles of the Declaration of Helsinki and approved by the Ethics Committee of Capital Medical University (grant number: 2020SY031). All participants provided written informed consent before taking part in this study.

### Data collection and definitions

Trained investigators collected data on sociodemographic characteristics, healthy lifestyles, disease history, and medication history through standard questionnaires. The questionnaires included age, sex, smoking status, drinking status, physical activity, the history of diseases, and medications. The medical history and medication history information of all participants were also linked to the electronic medical record system of the physical examination center. Smoking status was classified as “current smoker” and “nonsmoker.” Drinking status was classified as “current drinker” and “nondrinker.” Physical activity was defined as having moderate or intense exercise “≥20 minutes per time and ≥4 times per week.”^25^ The types of CVD included myocardial infarction and stroke. In addition, physical examination data were extracted from the medical record system. Body mass index was calculated as weight in kilograms divided by height in meters squared.^26^ Blood pressure was measured twice using a standard mercury sphygmomanometer after at least 10 minutes of rest on the right arm. Systolic blood pressure and diastolic blood pressure were recorded by the average of 2 measurements. Hypertension was defined as systolic blood pressure ≥140 mm Hg, diastolic blood pressure ≥90 mm Hg, the use of antihypertensive medication, or a history of hypertension.^27^

Fasting blood samples were analyzed using the automatic biochemical analyzer Beckman Coulter AU5800. The sdLDL-C concentration was quantified using a fully automated, 2-step homogeneous enzymatic catalase assay employing selective surfactants to isolate sdLDL-C (sdLDL-C Test Kit; Merit Choice Bioengineering; LOT: 190321).^28^ In the first step, proprietary surfactants selectively solubilize and degrade all serum lipoproteins except sdLDL, and cholesterol released from non-sdLDL particles is hydrolyzed by cholesterol esterase and cholesterol oxidase with concomitant H_2_O_2_ elimination by catalase. In the second step, the reaction solution was chemically modified to inhibit catalase activity (eg, by sodium azide). The remaining sdLDL in the sample was degraded and its cholesterol component released. The H_2_O_2_ produced during enzymatic hydrolysis of sdLDL-C then participated in the Trinder reaction, completing quantitative detection of sdLDL-C. The principle and performance of this surfactant-based homogeneous sdLDL-C assay have been described and validated previously.^28^ We directly measured triglyceride (TG) and total LDL-C concentrations by the enzymatic colorimetric method (GPO/PAP) and the surfactant assay method, as well as total cholesterol (TC) and high-density lipoprotein cholesterol (HDL-C) levels by the enzymatic method (Pureauto S CHO-N) and the catalase assay in the Beijing Health Management Cohort study. The lbLDL-C was calculated subtracting sdLDL-C from total LDL-C.^19^ We calculated sdLDL-C/LDL-C and sdLDL-C/lbLDL-C by dividing sdLDL-C by LDL-C and lbLDL-C, respectively. The non-HDL-C was calculated as TC minus HDL-C. Remnant cholesterol (RC) was estimated by subtracting LDL-C and HDL-C from TC.^29^

Several other blood parameters were also tested in the clinical laboratory of the physical examination center, including fasting blood glucose serum creatinine, uric acid, and high sensitivity C-reactive protein. Diabetes was defined as fasting blood glucose ≥7.0 mmol/L, the use of any antidiabetic medication, or previous history of diabetes.^30^ The estimated glomerular filtration rate was calculated from plasma creatinine using the Chronic Kidney Disease Epidemiology Collaboration creatinine equation.^31^ High sensitivity C-reactive protein was used to reflect the systemic inflammatory status.

### Definition of discordance

In this study, discordance was defined by using 2 approaches.^32^ First, residuals from linear regression models were used to reflect the difference between expected and measured sdLDL-C, lbLDL-C based on LDL-C or non-HDL-C. Discordance was categorized as sdLDL-C or lbLDL-C discordantly high (>75th percentile residual, observed sdLDL-C or lbLDL-C was much higher than expected), sdLDL-C or lbLDL-C discordantly low (<25th percentile residual, observed sdLDL-C or lbLDL-C was much lower than expected), or concordant (25th-75th percentile residual, observed sdLDL-C or lbLDL-C was similar to expected).

Second, we used medians to redefine discordance to verify the robustness of the results. Discordance was defined as sdLDL-C or lbLDL-C > median but the LDL-C or non-HDL-C ≤ its median, or vice versa. Taking discordance between sdLDL-C and LDL-C as an example, participants were divided into 4 groups: 1) both sdLDL-C and LDL-C ≤50th percentile; 2) sdLDL-C ≤50th percentile but LDL-C>50th percentile; 3) sdLDL-C >50th percentile but LDL-C ≤50th percentile; and 4) both sdLDL-C and LDL-C >50th percentile.

The discordance of the sdLDL-C/LDL-C ratio or sdLDL-C/lbLDL-C ratio with LDL-C, non-HDL-C, and sdLDL-C was also calculated using the same two methods.

### Definition of carotid plaque

Color Doppler ultrasound examinations of carotid artery were performed to diagnose carotid plaque by experienced radiologists using a high-resolution ultrasonography system (iU-22 ultrasound system; Philips Medical Systems), and the diastolic images were recorded for all the ultrasonographic images. Carotid intima-media thickness was quantified by the distance from the echo front of the lumen-intima to the echo front of the media adventitia, and was measured at 4 sites (right, left, near walls, and far walls) for the common carotid arteries, internal carotid artery, and carotid bifurcations according to a standardized protocol.^33^^,^^34^ The maximum carotid intima-media thickness of different carotid segments was used. Carotid plaque was defined as focal structures that encroach on the arterial lumen by at least 0.5 mm, 50% of the surrounding intima-media thickness value or carotid intima-media thickness >1.5 mm.^34^

### Statistical analysis

Baseline characteristics were presented as the mean ± SD, median (Q1-Q3), or number and percentage. We compared the differences in the characteristics according to the discordance between sdLDL-C and LDL-C groups. using 1-way analysis of variance test or Kruskal-Wallis test for continuous variables and chi-square test or Fisher exact test for categorical variables, as appropriate. Bonferroni correction was applied to correct for multiple testing. We also summarized the characteristics according to the presence of carotid plaque. The missing proportion and missing pattern of covariates with missing data are shown in Supplemental Figure 2. Specifically, the missing proportions for body mass index, systolic blood pressure, diastolic blood pressure, fasting blood glucose, high sensitivity C-reactive protein, uric acid, estimated glomerular filtration rate, smoking, drinking, and physical activity were 6.51% (n = 1,327 of 20,369), 5.58% (n = 1,137 of 20,369), 5.58% (n = 1,137 of 20,369), 0.01% (n = 3 of 20,369), 0.04% (n = 8 of 20,369), 0.01% (n = 3 of 20,369), 1.16% (n = 236 of 20,369), 21.41% (n = 4,360 of 20,369), 22.29% (n = 4,541 of 20,369), and 25.98% (n = 5,291 of 20,369), respectively. To prevent bias from the missing data mechanism, we used multiple imputation by chained equations to handle missing covariates in the main analyses.

We investigated the associations of sdLDL-C, lbLDL-C, sdLDL-C/LDL-C, and sdLDL-C/lbLDL-C (treated as both continuous and categorical variables) with the risk of carotid plaque using multivariable logistic regression models. To control for potential confounders, 2 models were established as follows: 1) model 1 was adjusted for age and sex; and 2) model 2 was further adjusted for body mass index, diabetes, hypertension, CVD, uric acid, estimated glomerular filtration rate, high sensitivity C-reactive protein, smoking status, drinking status, physical activity, the usage of antidiabetic medication, antihypertension medication, and lipid-lowering medication. Additionally, we adjusted for LDL-C, non-HDL-C, HDL-C, TC, TG, or RC in separate models to assess whether the observed associations remained independent of each lipid measure. Adjusted ORs and 95% CIs were obtained.

Then, using the same fully adjusted models, we performed discordance analyses to elucidate whether sdLDL-C, lbLDL-C, sdLDL-C/LDL-C ratio, and sdLDL-C/lbLDL-C ratio could provide more accurate insight into the risk of carotid plaque than LDL-C or non-HDL-C. The associations between sdLDL-C or lbLDL-C with LDL-C or non-HDL-C concordant/discordant groups and risk of carotid plaque were estimated based on difference in residual and medians. In addition, we assessed discordance in carotid plaque risk between the sdLDL-C/LDL-C and sdLDL-C/lbLDL-C ratios vs LDL-C, non-HDL-C, or sdLDL-C.

We used the area under the receiver-operating characteristic curve to assess model discrimination and decision curve analysis to evaluate clinical net benefit, before and after incorporation of sdLDL-C, lbLDL-C, the sdLDL-C/LDL-C ratio, and the sdLDL-C/lbLDL-C ratio into models containing LDL-C or non-HDL-C. Several sensitivity analyses were conducted to assess the robustness of our results. First, participants with the usage of antihypertensive medication, lipid-lowering medication, and antidiabetic medication were excluded. Second, participants with dyslipidemia were excluded. Dyslipidemia was defined as LDL-C ≥4.1 mmol/L, HDL-C <1.0 mmol/L, TG ≥2.3 mmol/L, TC≥6.2 mmol/L, any lipid-lowering medication, or self-reported dyslipidemia. Third, we repeated the analysis after excluding participants with CVD. Fourth, we reanalyzed the observed associations after excluding participants with missing covariate data. Fifth, given that plasma TG levels and RC are closely associated with LDL particle size and the proportion of sdLDL-C,^35^ we performed discordance analyses with further adjustment for TG or RC.

All analyses presented performed using Stata 16.0 (StataCorp) and R software (version 4.1.2; R Foundation for Statistical Computing). The difference was considered statistically significant at 2-sided *P* < 0.05.

## Results

### Baseline characteristics

Of the 20,369 participants, 51.28% (n = 10,445 of 20,369) were men, and the median age was 43.00 years (Q1-Q3: 34.00-54.00 years). Baseline population characteristics based on the discordance between sdLDL-C and LDL-C groups are presented in Table 1. Compared with concordant group, those with discordantly high sdLDL-C were more likely to be older and have CVD. They also had higher levels of sdLDL-C/LDL-C ratio, sdLDL-C/lbLDL-C ratio, TC, non-HDL-C, TG, RC, body mass index, systolic blood pressure, diastolic blood pressure, fasting blood glucose, and uric acid; had lower levels of HDL-C, lbLDL-C, and estimated glomerular filtration rate; tended to smoke, drink, be physically inactive, and use antidiabetic and antihypertensive medications. Characteristics according to the presence of carotid plaque are presented in Table 2. For the lipid profiles, only the difference in lbLDL-C levels between participants with and without carotid plaque was not statistically significant. Additionally, participants with carotid plaque tended to exhibit discordantly high sdLDL-C relative to LDL-C or non-HDL-C, discordantly low lbLDL-C relative to LDL-C or non-HDL-C, and discordantly high sdLDL-C/LDL-C or sdLDL-C/lbLDL-C relative to LDL-C, non-HDL-C, or sdLDL-C.

**Table 1:** Baseline Characteristics Among the Study Population Based on the Discordant of sdLDL-C With LDL-C

	Total (N = 20,369)	Discordant Low sdLDL-C (n = 5,092)	Concordant (n = 10,187)	Discordant High sdLDL-C (n = 5,090)	*P* Value
Age, y	43.00 (34.00-54.00)	39.00 (32.00-51.00)^a^	42.00 (34.00-55.00)	46.00 (38.00-56.00)^a^	<0.001
Male	10,445 (51.28)	1,299 (25.51)	5,263 (51.66)	3,883 (76.29)	<0.001
BMI, kg/m^2^	24.32 (21.95-26.79)	22.36 (20.43-24.54)	24.14 (21.98-26.50)	26.40 (24.45-28.78)^a^	<0.001
SBP, mm Hg	122.00 (112.00-134.00)	117.00 (108.00, 129.00)^a^	122.00 (111.00-134.00)	128.87 (118.00-139.00)^a^	<0.001
DBP, mm Hg	74.00 (66.00-81.00)	70.00 (64.00-77.00)^a^	73.00 (66.00-80.63)	78.00 (72.00-86.00)^a^	<0.001
sdLDL-C, mmol/L	0.90 (0.64-1.20)	0.62 (0.50-0.78)^a^	0.87 (0.67-1.08)	1.33 (1.15-1.53)^a^	<0.001
sbLDL-C, mmol/L	1.95 (1.60-2.35)	2.32 (1.97-2.73)^a^	1.88 (1.54-2.25)	1.75 (1.41-2.10)^a^	<0.001
sdLDL-C/LDL-C ratio	0.31 (0.25-0.38)	0.22 (0.19-0.24)^a^	0.31 (0.28-0.35)	0.42 (0.40-0.46)^a^	<0.001
sdLDL-C/lbLDL-C ratio	0.46 (0.33-0.61)	0.28 (0.24-0.31)^a^	0.46 (0.39-0.53)	0.74 (0.66-0.86)^a^	<0.001
TG, mmol/L	1.09 (0.75-1.64)	0.72 (0.57-0.90)^a^	1.05 (0.78-1.36)	2.13 (1.66-2.78)^a^	<0.001
RC, mmol/L	0.68 (0.53-0.89)	0.61 (0.49-0.74)^a^	0.62 (0.49-0.78)	0.99 (0.79-1.27)^a^	<0.001
TC, mmol/L	4.97 (4.36-5.62)	5.20 (4.67-5.80)^a^	4.69 (4.09-5.35)	5.24 (4.66-5.87)^a^	<0.001
LDL-C, mmol/L	2.90 (2.38-3.44)	2.97 (2.49-3.49)^a^	2.75 (2.23-3.31)	3.09 (2.59-3.58)^a^	<0.001
HDL-C, mmol/L	1.28 (1.09-1.52)	1.59 (1.41-1.80)^a^	1.28 (1.12-1.46)	1.05 (0.93-1.19)^a^	<0.001
Non–HDL-C, mmol/L	3.64 (3.01-4.30)	3.57 (3.06-4.18)^a^	3.38 (2.76-4.06)	4.15 (3.62-4.76)^a^	<0.001
FBG, mmol/L	5.06 (4.77-5.44)	4.89 (4.65-5.18)^a^	5.05 (4.77-5.41)	5.30 (4.97-5.82)^a^	<0.001
High-sensitivity C-reactive protein, mmol/L	1.00 (0.17-2.14)	0.78 (0.02-1.89)^a^	0.94 (0.15-2.05)	1.37 (0.42-2.53)	<0.001
UA, mmol/L	332.00 (273.00-402.00)	287.00 (245.00-341.00)^a^	329.00 (273.00-395.00)	390.00 (332.00-452.00)^a^	<0.001
eGFR, mL/min/1.73 m^2^	109.08 (99.88-117.68)	111.95 (102.67-120.09)^a^	109.23 (99.59-117.94)	106.49 (98.44-114.19)^a^	<0.001
Current smoker	3,162 (15.52)	302 (5.93)^a^	1,467 (14.40)	1,393 (27.37)^a^	<0.001
Current drinker	1,586 (7.79)	122 (2.40)^a^	704 (6.91)	760 (14.93)^a^	<0.001
Physical activity	6,998 (34.36)	1,788 (35.11)	3,552 (34.87)	1,658 (32.57)^a^	<0.001
Antidiabetic medication use	505 (2.48)	44 (0.86)^a^	275 (2.70)	186 (3.65)^a^	<0.001
Antihypertension medication use	1,477 (7.25)	130 (2.55)^a^	750 (7.36)	597 (11.73)^a^	<0.001
Lipid-lowering medication use	655 (3.22)	65 (1.28)^a^	373 (3.66)	217 (4.26)	<0.001
Diabetes	1,316 (6.46)	121 (2.38)^a^	584 (5.73)	611 (12.00)^a^	<0.001
Hypertension	4,855 (23.84)	684 (13.43)^a^	2,344 (23.01)	1,827 (35.89)^a^	<0.001
Dyslipidemia	6,450 (31.67)	866 (17.01)^a^	2,166 (21.26)	3,418 (67.15)^a^	<0.001
Carotid plaque	5,508 (27.04)	894 (17.56)^a^	2,732 (26.82)	1,882 (36.97)^a^	<0.001
CVD	1,484 (7.29)	250 (4.91)^a^	736 (7.22)	498 (9.78)^a^	<0.001

Values are median (Q1-Q3) or n (%). Characteristics of the study population were compared using 1-way analysis of variance test or Kruskal-Wallis test for continuous variables and the chi-square test or Fisher exact test for categorical variables. Discordance was defined by the residuals of expected and measured sdLDL-C based on LDL-C through linear regression models. sdLDL-C was discordantly low (<25th percentile residual), concordant (25th-75th percentiles residual), or discordantly high (>75th percentile residual).

BMI = body mass index; CVD = cardiovascular diseases; DBP = diastolic blood pressure; eGFR = estimated glomerular filtration rate; FBG = fasting blood glucose; HDL-C = high-density lipoprotein cholesterol; lbLDL-C = large buoyant low-density lipoprotein cholesterol; LDL-C = low-density lipoprotein cholesterol; RC = remnant cholesterol; SBP = systolic blood pressure; sdLDL-C = small dense low-density lipoprotein cholesterol; TC = total cholesterol; TG = triglyceride; UA = uric acid.

a Bonferroni adjusted *P* values of <0.05 compared with reference group (concordant).

**Table 2:** Baseline Characteristics Among the Study Population Based on the Presence of Carotid Plaque

	Total (N = 20,369)	Population Without Carotid Plaque (n = 14,861)	Population With Carotid Plaque (n = 5,508)	*P* Value
Age, y	43.00 (34.00-54.00)	39.00 (32.00-48.00)	57.00 (48.00-64.00)	<0.001
Male	10,445 (51.28)	6,819 (45.89)	3,626 (65.83)	<0.001
BMI, kg/m^2^	24.32 (21.95-26.79)	23.88 (21.52-26.52)	25.20 (23.18-27.42)	<0.001
SBP, mm Hg	122.00 (112.00-134.00)	120.00 (110.00-131.00)	131.00 (119.00-143.00)	<0.001
DBP, mm Hg	74.00 (66.00-81.00)	72.00 (65.00-80.00)	77.00 (71.00-86.00)	<0.001
sdLDL-C, mmol/L	0.90 (0.64-1.20)	0.85 (0.61-1.15)	1.03 (0.76-1.31)	<0.001
lbLDL-C, mmol/L	1.95 (1.60-2.35)	1.95 (1.61-2.33)	1.97 (1.54-2.40)	0.807
sdLDL-C/LDL-C ratio	0.31 (0.25-0.38)	0.30 (0.24-0.37)	0.35 (0.29-0.40)	<0.001
SdLDL-C/lbLDL-C ratio	0.46 (0.33-0.61)	0.43 (0.32-0.58)	0.53 (0.40-0.67)	<0.001
TG, mmol/L	1.09 (0.75-1.64)	1.01 (0.70-1.55)	1.30 (0.92-1.85)	<0.001
RC, mmol/L	0.68 (0.53-0.89)	0.66 (0.51-0.85)	0.77 (0.60-0.98)	<0.001
TC, mmol/L	4.97 (4.36-5.62)	4.92 (4.34-5.55)	5.13 (4.45-5.82)	<0.001
LDL-C, mmol/L	2.90 (2.38-3.44)	2.85 (2.37-3.38)	3.02 (2.43-3.60)	<0.001
HDL-C, mmol/L	1.28 (1.09-1.52)	1.31 (1.11-1.55)	1.22 (1.04-1.44)	<0.001
Non-HDL-C, mmol/L	3.64 (3.01-4.30)	3.57 (2.96-4.21)	3.87 (3.17-4.55)	<0.001
FBG, mmol/L	5.06 (4.77-5.44)	4.98 (4.72-5.30)	5.34 (4.98-5.93)	<0.001
High-sensitivity C-reactive protein, mmol/L	1.00 (0.17-2.14)	0.94 (0.14-2.07)	1.14 (0.32-2.34)	<0.001
UA, mmol/L	332.00 (273.00-402.00)	327.00 (267.00-399.00)	347.00 (291.00-408.00)	<0.001
eGFR, mL/min/1.73 m^2^	109.08 (99.88-117.68)	112.53 (104.18-119.91)	99.83 (92.30-107.02)	<0.001
Current smoker	3,162 (15.52)	1,929 (12.98)	1,233 (22.39)	<0.001
Current drinker	1,586 (7.79)	846 (5.69)	740 (13.44)	<0.001
Physical activity n (%)	6,998 (34.36)	4,521 (30.42)	2,477 (44.97)	<0.001
Antidiabetic medication use	505 (2.48)	169 (1.14)	336 (6.10)	<0.001
Antihypertension medication use	1,477 (7.25)	608 (4.09)	869 (15.78)	<0.001
Lipid-lowering medication use	655 (3.22)	207 (1.39)	448 (8.13)	<0.001
Diabetes	1,316 (6.46)	465 (3.13)	851 (15.45)	<0.001
Hypertension	4,855 (23.84)	2,445 (16.45)	2,410 (43.75)	<0.001
Dyslipidemia	6,450 (31.67)	4,013 (27.00)	2,437 (44.24)	< 0.001
CVD	1,484 (7.29)	849 (5.71)	635 (11.53)	<0.001
Discordant groups				
sdLDL-C and LDL-C				<0.001
Discordant low sdLDL-C	5,092 (25.00)	4,198 (28.25)	894 (16.23)	
Concordant	10,187 (50.01)	7,455 (50.16)	2,732 (49.60)	
Discordant high sdLDL-C	5,090 (24.99)	3,208 (21.59)	1,882 (34.17)	
sdLDL-C and non-HDL-C				<0.001
Discordant low sdLDL-C	5,092 (25.00)	4,085 (27.49)	1,007 (18.28)	
Concordant	10,185 (50.00)	7,483 (50.35)	2,702 (49.06)	
Discordant high sdLDL-C	5,092 (25.00)	3,293 (22.16)	1,799 (32.66)	
lbLDL-C and LDL-C				<0.001
Discordant low lbLDL-C	5,093 (25.00)	3,209 (21.59)	1,884 (34.20)	
Concordant	10,183 (49.99)	7,454 (50.16)	2,729 (49.55)	
Discordant high lbLDL-C	5,093 (25.00)	4,198 (28.25)	895 (16.25)	
lbLDL-C and non-HDL-C				<0.001
Discordant low lbLDL-C	5,093 (25.00)	3,190 (21.47)	1,903 (34.55)	
Concordant	10,183 (49.99)	7,625 (51.31)	2,558 (46.44)	
Discordant high lbLDL-C	5,093 (25.00)	4,046 (27.23)	1,047 (19.01)	
sdLDL-C/LDL-C and LDL-C				<0.001
Discordant low sdLDL-C/LDL-C	5,094 (25.01)	4,277 (28.78)	817 (14.83)	
Concordant	10,182 (49.99)	7,377 (49.64)	2,805 (50.93)	
Discordant high sdLDL-C/LDL-C	5,093 (25.00)	3,207 (21.58)	1,886 (34.24)	
sdLDL-C/LDL-C and non-HDL-C				<0.001
Discordant low sdLDL-C/LDL-C	5,093 (25.00)	4,147 (27.91)	946 (17.18)	
Concordant	10,183 (50.00)	7,475 (50.30)	2,708 (49.16)	
Discordant high sdLDL-C/LDL-C	5,092 (25.00)	3,349 (22.54)	1,743 (31.64)	
sdLDL-C/LDL-C and sdLDL-C				<0.001
Discordant low sdLDL-C/LDL-C	5,094 (25.01)	3,746 (25.21)	1,348 (24.47)	
Concordant	10,183 (49.99)	7,766 (52.26)	2,417 (43.88)	
Discordant high sdLDL-C/LDL-C	5,092 (25.00)	3,349 (22.54)	1,743 (31.64)	
sdLDL-C/lbLDL-C and LDL-C				<0.001
Discordant low sdLDL-C/lbLDL-C	5,092 (25.00)	4,322 (29.08)	770 (13.98)	
Concordant	10,184 (50.00)	7,331 (49.33)	2,853 (51.81)	
Discordant high sdLDL-C/lbLDL-C	5,093 (25.00)	3,208 (21.59)	1,885 (34.22)	
sdLDL-C/lbLDL-C and non-HDL-C				<0.001
Discordant low sdLDL-C/lbLDL-C	5,093 (25.00)	4,085 (27.49)	1,008 (18.30)	
Concordant	10,183 (50.00)	7,550 (50.80)	2,633 (47.81)	
Discordant high sdLDL-C/lbLDL-C	5,093 (25.00)	3,226 (21.71)	1,867 (33.90)	
sdLDL-C/lbLDL-C and sdLDL-C				<0.001
Discordant low sdLDL-C/lbLDL-C	5,092 (25.00)	3,533 (23.77)	1,559 (28.31)	
Concordant	10,185 (50.00)	7,950 (53.50)	2,235 (40.58)	
Discordant high sdLDL-C/lbLDL-C	5,092 (25.00)	3,378 (22.73)	1,714 (31.12)	

Values are median (Q1-Q3) or n (%). Characteristics of the study population were compared using Student’s *t*-test or Mann-Whitney *U* test for continuous variables and the chi-square test or Fisher exact test for categorical variables.

Abbreviations as in Table 1.

### Association of lipid profiles with carotid plaque

As displayed in Table 3, we observed significant associations of higher sdLDL-C, lbLDL-C, sdLDL-C/LDL-C ratio, and sdLDL-C/lbLDL-C ratio with the increased risks of carotid plaque. In model 2, the adjusted ORs for carotid plaque risk were 1.354 (95% CI: 1.299-1.410) for sdLDL-C per 1-SD increase, 1.110 (95% CI: 1.070-1.151) for lbLDL-C per 1-SD increase, 1.196 (95% CI: 1.148-1.247) for sdLDL-C/LDL-C ratio per 1-SD increase, and 1.153 (95% CI: 1.110-1.197) for sdLDL-C/lbLDL-C ratio per 1-SD increase, respectively. Similar results were observed when these lipid parameters were treated as categorical variables.

**Table 3:** Associations of sdLDL-C, lbLDL-C, sdLDL-C/LDL-C, and sdLDL-C/lbLDL-C With Carotid Plaque

	Model 1		Model 2	
	OR (95% CI)	*P* Value	OR (95% CI)	*P* Value
sdLDL-C, mmol/L				
Categorical				
Quartile 1	Reference		Reference	
Quartile 2	1.354 (1.212-1.513)	<0.001	1.315 (1.168-1.480)	<0.001
Quartile 3	1.664 (1.491-1.856)	<0.001	1.594 (1.416-1.793)	<0.001
Quartile 4	2.301 (2.065-2.565)	<0.001	2.204 (1.953-2.487)	<0.001
Per 1-SD increase	1.361 (1.312-1.411)	<0.001	1.354 (1.299-1.410)	<0.001
lbLDL-C, mmol/L				
Categorical				
Quartile 1	Reference		Reference	
Quartile 2	0.808 (0.731-0.893)	<0.001	0.913 (0.820-1.017)	0.099
Quartile 3	0.931 (0.844-1.027)	0.152	1.057 (0.951-1.174)	0.304
Quartile 4	1.185 (1.076-1.304)	0.001	1.261 (1.136-1.398)	<0.001
Per 1-SD increase	1.080 (1.043-1.118)	<0.001	1.110 (1.070-1.151)	<0.001
sdLDL-C/LDL-C				
Categorical				
Quartile 1	Reference		Reference	
Quartile 2	1.139 (1.018-1.274)	0.024	1.105 (0.982-1.243)	0.098
Quartile 3	1.727 (1.548-1.926)	<0.001	1.558 (1.387-1.751)	<0.001
Quartile 4	2.066 (1.850-2.307)	<0.001	1.778 (1.572-2.011)	<0.001
Per 1-SD increase	1.270 (1.225-1.318)	<0.001	1.196 (1.148-1.247)	<0.001
sdLDL-C/lbLDL-C				
Categorical				
Quartile 1	Reference		Reference	
Quartile 2	1.135 (1.015-1.271)	0.027	1.103 (0.980-1.241)	0.105
Quartile 3	1.718 (1.540-1.916)	<0.001	1.551 (1.380-1.744)	<0.001
Quartile 4	2.071 (1.855-2.313)	<0.001	1.782 (1.576-2.015)	<0.001
Per 1-SD increase	1.216 (1.176-1.258)	<0.001	1.153 (1.110-1.197)	<0.001

Model 1 was adjusted for age and sex. Model 2 was adjusted for age, sex, diabetes, hypertension, cardiovascular diseases, UA, eGFR, high-sensitivity C-reactive protein, smoking status, drinking status, body mass index, the usage of antidiabetic medication, antihypertension medication, lipid-lowering medication, and physical activity.

Abbreviations as in Table 1.

Other traditional lipid parameters, including LDL-C, non-HDL-C, HDL-C, TC, TG, and RC, were also associated with carotid plaque risk (Supplemental Table 1). However, after further adjustment for these parameters, sdLDL-C, sdLDL-C/LDL-C ratio, and sdLDL-C/lbLDL-C ratio remained positively associated with carotid plaque risk (Supplemental Table 2). In contrast, the association between lbLDL-C and carotid plaque became inversely correlated after adjustment for LDL-C, TC, and non-HDL-C.

### Discordance analysis of sdLDL-C and lbLDL-C

We performed discordant analyses of sdLDL-C and lbLDL-C vs LDL-C and non-HDL-C to investigate which of sdLDL-C or lbLDL-C is more closely related to the risk of carotid plaque than LDL-C and non-HDL-C. As shown in Supplemental Figure 3, among the 20,369 participants, the proportions of participants with carotid plaque in discordantly low sdLDL-C (17.56% [n = 894 of 5,092] vs LDL-C; 19.78% [n = 1,007 of 5,092] vs non-HDL-C), concordant (26.82% [n = 2,732 of 10,187] vs LDL-C; 26.53% [n = 2,702 of 10,185] vs non-HDL-C), and discordantly high sdLDL-C (36.97% [n = 1,882 of 5,090] vs LDL-C; 35.33% [n = 1,799 of 5,092] vs non-HDL-C)] vs LDL-C or non-HDL-C groups were gradually increased (Supplemental Figures 3A and 3C). Conversely, the proportion of participants with carotid plaque decreased gradually from the discordantly low lbLDL-C (36.99% [n = 1,884 of 5,093] vs LDL-C; 37.37% [n = 1,903 of 5,093] vs non-HDL-C), to the concordant (26.80% [n = 2,729 of 10,183] vs LDL-C; 25.12% [n = 2,558 of 10,183] vs non-HDL-C), and then to the discordantly high lbLDL-C (17.57% [n = 895 of 5,093] vs LDL-C; 20.56% [n = 1,047 of 5,093] vs non-HDL-C) vs LDL-C or non-HDL-C groups (Supplemental Figures 3B and 3D). The differences in the previous proportions were all significant (Bonferroni correction *P* < 0.001).

**Figure 1:**
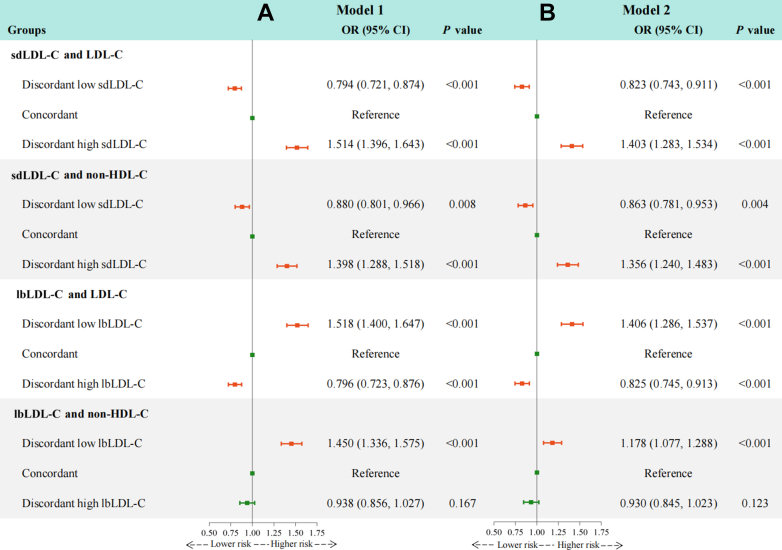
Discordance Analyses of sdLDL-C and lbLDL-C According to Residual Differences The figure shows the associations between discordant small dense low-density lipoprotein cholesterol (sdLDL-C), large buoyant low-density lipoprotein cholesterol (lbLDL-C) relative to low-density lipoprotein cholesterol (LDL-C) or non–high-density lipoprotein cholesterol (non-HDL-C) and the risk of carotid plaque, based on residual differences, using logistic regression after adjusting for potential confounders. (A) Model 1 was adjusted for age and sex. (B) Model 2 was further adjusted for body mass index, diabetes, hypertension, cardiovascular disease, uric acid, estimated glomerular filtration rate, high-sensitivity C-reactive protein, smoking status, drinking status, use of antidiabetic medication, antihypertensive medication, lipid-lowering medication, and physical activity. Discordant low sdLDL-C or lbLDL-C was defined as <25th percentile residual, concordant as 25th to 75th percentile residual, and discordant high sdLDL-C or lbLDL-C as >75th percentile residual. Orange error bars indicate statistically significant OR values, while green error bars indicate non-significant OR values.

We first used residuals to define discordance and presented the results in Figure 1. In the fully adjusted model, individuals with discordantly high sdLDL-C relative to LDL-C (OR: 1.403; 95% CI: 1.283-1.534) or non-HDL-C (OR: 1.356; 95% CI: 1.240-1.483) had a higher risk of carotid plaque compared with those in the concordant group. In contrast, discordantly low sdLDL-C vs LDL-C (OR: 0.823; 95% CI: 0.743-0.911) or non-HDL-C (OR: 0.863; 95% CI: 0.781-0.953) was associated with lower odds of carotid plaque. Using concordant lbLDL-C with LDL-C or non-HDL-C as the reference, discordantly high lbLDL-C relative to LDL-C (OR: 0.825; 95% CI: 0.745-0.913) was significantly associated with a decreased risk of carotid plaque, while discordantly low lbLDL-C relative to LDL-C (OR: 1.406; 95% CI: 1.286-1.537) or non-HDL-C (OR: 1.178; 95% CI: 1.077-1.288) was significantly associated with an increased risk.

**Figure 2:**
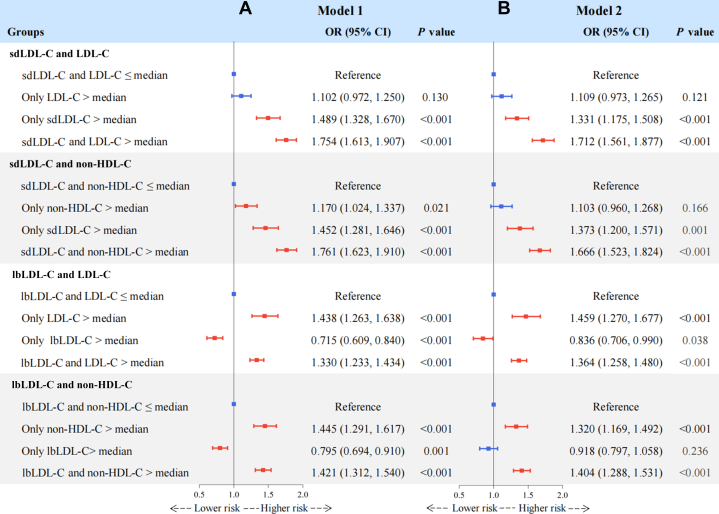
Discordance Analyses of sdLDL-C and lbLDL-C According to Medians The figure shows the associations between discordant sdLDL-C, lbLDL-C relative to LDL-C, or non-HDL-C and the risk of carotid plaque, based on medians, using logistic regression after adjusting for potential confounders. (A) Model 1 was adjusted for age and sex. (B) Model 2 was further adjusted for body mass index, diabetes, hypertension, cardiovascular disease, uric acid, estimated glomerular filtration rate, high-sensitivity C-reactive protein, smoking status, drinking status, use of antidiabetic medication, antihypertensive medication, lipid-lowering medication, and physical activity. Discordance was defined as sdLDL-C or lbLDL-C > median but the LDL-C or non-HDL-C ≤ its median, or vice versa. Red error bars indicate statistically significant OR values, while blue error bars indicate nonsignificant OR values. Abbreviations as in Figure 1.

Then, we redefined discordance based on medians and divided the population into 4 groups to validate the robustness of our findings (Figure 2). Compared with individuals with both sdLDL-C and LDL-C or non-HDL-C ≤ median, those with “only sdLDL-C > median but LDL-C or non-HDL-C ≤ median,” rather than “only LDL-C or non-HDL-C > median but sdLDL-C ≤ median” had significantly higher risks of carotid plaque. Conversely, for the discordance between lbLDL-C and LDL-C or non-HDL-C (Figure 2), group with “lbLDL-C ≤ median but LDL-C or non-HDL-C > median” showed significantly increased risk of carotid plaque.

### Discordance analysis of sdLDL-C/LDL-C and sdLDL-C/lbLDL-C ratios

We also conducted discordance analyses of the sdLDL-C/LDL-C ratio and sdLDL-C/lbLDL-C ratio vs LDL-C, non-HDL-C, or sdLDL-C to further elucidate whether these ratios can identify a high-risk carotid plaque population not detected by sdLDL-C and other traditional lipid parameters. The percentage of participants with carotid plaque (Supplemental Figure 4) was highest in the discordantly high sdLDL-C/LDL-C ratio or sdLDL-C/lbLDL-C ratio relative to LDL-C, non-HDL-C or sdLDL-C groups, compared with the discordantly low and concordant groups (Bonferroni correction *P <* 0.001).

**Figure 3:**
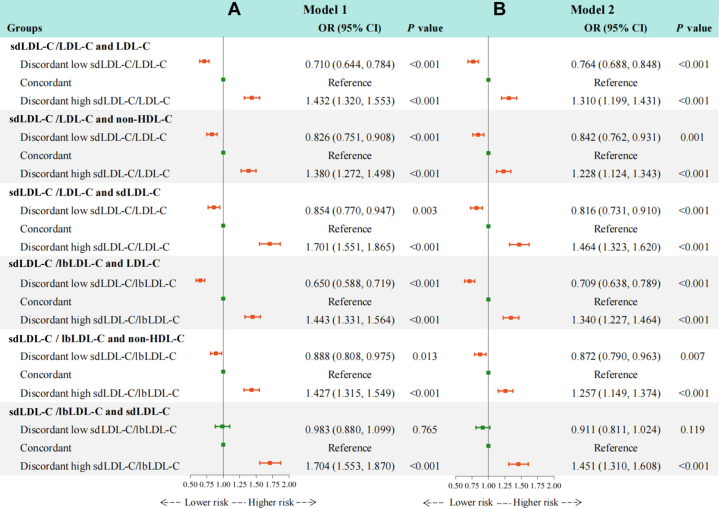
Discordance Analyses of sdLDL-C/LDL-C and sdLDL-C/lbLDL-C According to Residual Differences The figure shows the associations of discordant sdLDL-C/LDL-C and sdLDL-C/lbLDL-C ratios relative to LDL-C, non-HDL-C, or sdLDL-C with the risk of carotid plaque, based on residual differences, using logistic regression after adjusting for potential confounders. (A) Model 1 adjusted for age and sex. (B) Model 2 further adjusted for body mass index, diabetes, hypertension, cardiovascular disease, uric acid, estimated glomerular filtration rate, high-sensitivity C-reactive protein, smoking status, drinking status, use of antidiabetic medication, antihypertensive medication, lipid-lowering medication, and physical activity. Discordant low sdLDL-C/LDL-C or sdLDL-C/lbLDL-C ratio was defined as <25th percentile residual, concordant as 25th to 75th percentile residual, and discordant high sdLDL-C/LDL-C or sdLDL-C/lbLDL-C ratio as >75th percentile residual. Orange error bars indicate statistically significant OR values, while green error bars indicate nonsignificant OR values. Abbreviations as in Figure 1.

**Figure 4:**
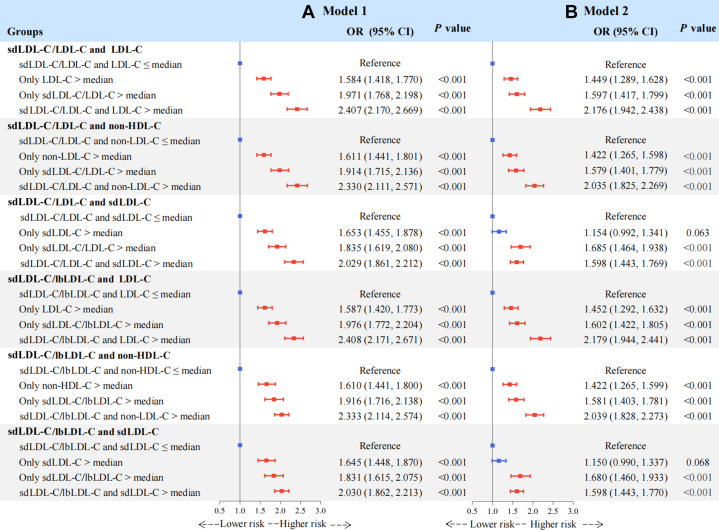
Discordance Analyses of sdLDL-C/LDL-C and sdLDL-C/lbLDL-C According to Medians The figure shows the associations of discordant sdLDL-C/LDL-C and sdLDL-C/lbLDL-C ratios relative to LDL-C, non-HDL-C, or sdLDL-C with the risk of carotid plaque, based on medians, using logistic regression after adjusting for potential confounders. (A) Model 1 was adjusted for age and sex. (B) Model 2 was further adjusted for body mass index, diabetes, hypertension, cardiovascular disease, uric acid, estimated glomerular filtration rate, high-sensitivity C-reactive protein, smoking status, drinking status, use of antidiabetic medication, antihypertensive medication, lipid-lowering medication, and physical activity. Discordance was defined as sdLDL-C/LDL-C ratio or sdLDL-C/lbLDL-C ratio > median but the LDL-C, non-HDL-C ,or sdLDL-C ≤ its median, or vice versa. Red error bars indicate statistically significant OR values, while blue error bars indicate nonsignificant OR values. Abbreviations as in Figure 1.

Using residual defined categories (Figure 3), discordantly low sdLDL-C/LDL-C ratios relative to LDL-C (OR: 0.764; 95% CI: 0.688-0.848), non-HDL-C (OR: 0.842; 95% CI: 0.762-0.931), and sdLDL-C (OR: 0.816; 95% CI: 0.731-0.910) were associated with significantly lower risks of carotid plaque compared with individuals with concordant levels. Conversely, sdLDL-C/LDL-C ratios discordantly higher than LDL-C (OR: 1.310; 95% CI: 1.199-1.431), non-HDL-C (OR: 1.228; 95% CI: 1.124-1.343), and sdLDL-C (OR: 1.464; 95% CI: 1.323-1.620) were associated with significantly higher risks of carotid plaque. Similarly, the discordantly low sdLDL-C/lbLDL-C ratio relative to LDL-C, non-HDL-C, and sdLDL-C was also negatively associated with carotid plaque risk, whereas the discordantly high ratio was positively associated with the risk. Furthermore, as shown in Figure 4, we performed a supplementary analysis based on medians, which showed that individuals with “only sdLDL-C/LDL-C ratio or sdLDL-C/lbLDL-C ratio > median” had a higher OR for carotid plaque than those with “only LDL-C, non-HDL-C, or sdLDL-C > median.”

### Additional analyses

Incorporating sdLDL-C, lbLDL-C, sdLDL-C/LDL-C ratio, or sdLDL-C/lbLDL-C ratio into the model enhances the predictive ability for carotid plaque, compared with using traditional indicators such as LDL-C (Supplemental Figure 5) or non-HDL-C (Supplemental Figure 6) alone. Notably, the inclusion of sdLDL-C, sdLDL-C/LDL-C ratio, and sdLDL-C/lbLDL-C ratio in the model that incorporated non-HDL-C resulted in significantly higher area under the receiver-operating characteristic curve values compared with the inclusion of lbLDL-C (*P* < 0.05). Supplemental Figures 7 and 8 present the results of the decision curve analysis. When the threshold probability ranged from 0.05 to 0.85, incorporating sdLDL-C, sdLDL-C/LDL-C ratio, and sdLDL-C/lbLDL-C ratio into the models that include LDL-C (Supplemental Figure 7) or non-HDL-C (Supplemental Figure 8) slightly improved the net benefit compared with the original models. However, no such improvement was observed with the addition of lbLDL-C to the models.

When we excluded participants using antidiabetic, antihypertensive, and lipid-lowering medications (n = 18,396) (Supplemental Table 3 and 4) or those with dyslipidemia (n = 13,919) (Supplemental Tables 5 and 6), the results did not change substantially. Furthermore, the results remained consistent after excluding participants with CVD (n = 18,885) (Supplemental Tables 7 and 8), excluding those with missing covariate data (n = 13,832) (Supplemental Tables 9 and 10), or adjusting for TG and RC (Supplemental Tables 11 and 12).

## Discussion

In this study, we found that, first, higher levels of sdLDL-C, sdLDL-C/LDL-C ratio, and sdLDL-C/lbLDL-C ratio were more strongly associated with an increased risk of carotid plaque than lbLDL-C. After further adjustment for LDL-C, HDL-C, TC, non-HDL-C, TG, and RC, the positive associations for sdLDL-C, sdLDL-C/LDL-C ratio, and sdLDL-C/lbLDL-C ratio remained significant but not for lbLDL-C. Second, discordantly high sdLDL-C and discordantly low lbLDL-C relative to LDL-C or non-HDL-C were significantly correlated with an elevated risk of carotid plaque, compared with the concordance groups. In contrast, groups with discordantly low sdLDL-C and discordantly high lbLDL-C vs LDL-C or non-HDL-C had a reduced risk (Central Illustration). Third, the discordance analyses of sdLDL-C/LDL-C ratio, sdLDL-C/lbLDL-C ratio vs LDL-C, non-HDL-C, and sdLDL-C suggest that even at low concentrations of LDL-C, non-HDL-C, or sdLDL-C, a high sdLDL-C/LDL-C ratio or sdLDL-C/lbLDL-C ratio remains atherogenic (Central Illustration). These findings highlight the importance of monitoring sdLDL-C levels and calculating the sdLDL-C/LDL-C and sdLDL-C/lbLDL-C ratios to optimize residual risk assessment and intervention strategies for atherosclerosis in routine clinical practice.

**Central Illustration:**
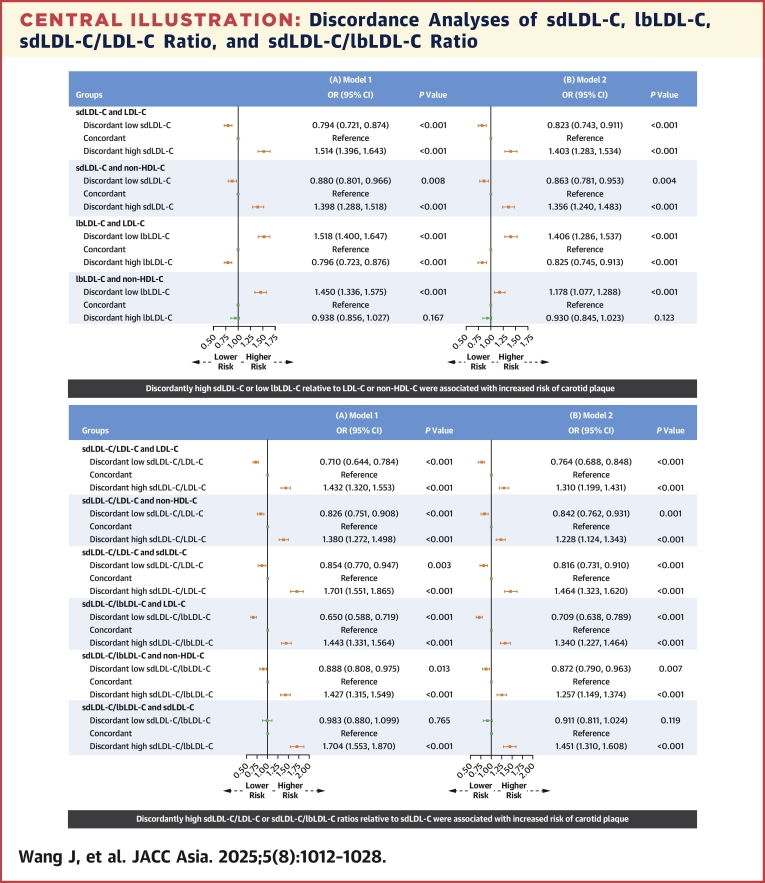
Discordance Analyses of sdLDL-C, lbLDL-C, sdLDL-C/LDL-C Ratio, and sdLDL-C/lbLDL-C Ratio Model 1 was adjusted for age and sex. Model 2 was further adjusted for body mass index, diabetes, hypertension, cardiovascular disease, uric acid, estimated glomerular filtration rate, high-sensitivity C-reactive protein, smoking status, drinking status, use of antidiabetic medication, antihypertensive medication, lipid-lowering medication, and physical activity. Discordant low was defined as <25th percentile residual, concordant as 25th to 75th percentile residual, and discordant high as >75th percentile residual. Orange error bars indicate statistically significant OR values, while green error bars indicate non-significant OR values. HDL-C = high-density lipoprotein cholesterol; lbLDL-C = large buoyant low-density lipoprotein cholesterol; LDL-C = low-density lipoprotein cholesterol; sdLDL-C = small dense low-density lipoprotein cholesterol.

Several studies have demonstrated that sdLDL-C was associated with CVD.^36^ A recent study showed that higher levels of sdLDL-C, rather than lbLDL-C, were related to an increased risk of ischemic stroke.^19^ Atherosclerosis is known as the primary pathological basis for CVD. However, prior studies on the association between sdLDL-C and carotid atherosclerosis have been limited by small sample sizes and non-representative populations, and no studies have focused on lbLDL-C, sdLDL-C/LDL-C ratio, and sdLDL-C/lbLDL-C ratio. A study involving 887 participants identified sdLDL-C as an independent risk factor for carotid plaque.^37^ Our study provided epidemiological evidence to complement related research based on a general population with a large sample size, revealing for the first time that the risk for carotid plaque is more strongly associated with sdLDL-C than lbLDL-C. In addition, we further emphasize the importance of considering the proportion of sdLDL-C within total LDL-C and the sdLDL-C/lbLDL-C ratio when assessing the risk of carotid plaque. After adjusting for multiple atherogenic lipid components, sdLDL-C, sdLDL-C/LDL-C, and sdLDL-C/lbLDL-C remain robust predictors of carotid plaque risk. However, after adjusting for LDL-C, TC, or non-HDL-C, lbLDL-C showed an inverse association with carotid plaque. These findings suggest that sdLDL, rather than lbLDL, may be the principal LDL subfraction contributing to atherosclerotic plaque formation.

In clinical practice, LDL-C has been the main target of lipid-lowering therapy. Despite lowering LDL-C levels, patients still experience high residual cardiovascular risk, prompting increased interest in the distinct effects of LDL subtypes. To date, it remains uncertain whether sdLDL-C, lbLDL-C, or their ratio indices can better reflect CVD risk beyond LDL-C or non-HDL-C. The Prospective Framingham Offspring Study showed that sdLDL-C more accurately reflected CVD risk beyond non-HDL-C,^20^ whereas another study found no evidence that sdLDL-C was superior to LDL-C in assessing coronary heart disease risk among individuals with normal fasting glucose.^21^ In our study, we used 2 approaches, including residual difference and median methods, to define discordance, and focused on the subclinical stage of CVD. We confirmed that discordantly higher sdLDL-C relative to LDL-C or non-HDL-C was associated with increased carotid plaque risk, whereas discordantly lower sdLDL-C conferred reduced risk. This finding suggests that sdLDL-C contributes to carotid plaque development beyond total LDL-C and non-HDL-C. Conversely, discordantly elevated lbLDL-C was associated with reduced plaque risk, whereas discordantly low lbLDL-C correlated with increased risk, suggesting that lbLDL-C follows an atherogenic pattern distinct from other LDL subfractions, with implications for personalized lipid management. Clinically, in patients who have achieved low LDL-C and non-HDL-C levels, elevated lbLDL-C may not require the same level of therapeutic intervention, potentially reducing the burden of unnecessary lipid-lowering treatment. However, persistent elevation of sdLDL-C after treatment may still require intensified lipid lowering. Furthermore, approaches that shift the LDL-C distribution toward a higher proportion of lbLDL-C during LDL-C and non-HDL-C–lowering therapy may confer additional cardiovascular benefit. Our findings also suggest that the sdLDL-C/LDL-C and sdLDL-C/lbLDL-C ratios offer clinical insight beyond sdLDL-C alone. Routine monitoring of these ratios may enable a more personalized approach to residual CVD risk management at early stages, especially in patients who achieve normal LDL-C and non-HDL-C levels with standard lipid-lowering therapy. These novel findings underscore the need for a nuanced approach to lipid therapy and management, focusing more on LDL subtype distribution rather than solely on total lipid reduction, thereby optimizing patient care and clinical outcomes while minimizing overtreatment.

Some mechanisms may explain the previous associations. LDL particles are heterogeneous, comprising particles varying in size, density, and chemical composition. sdLDL particles are smaller and denser,^16^ while lbLDL particles are larger and less dense.^38^ These characteristics make sdLDL particles more likely to penetrate the arterial intima compared with lbLDL particles. Additionally, sdLDL exhibits reduced LDL receptor affinity, resulting in slower plasma clearance and prolonged half-life, which enhances sdLDL deposition in the vessel wall and promotes atherosclerotic plaque formation.^39^ By contrast, lbLDL particles confer higher LDL receptor affinity and undergo more rapid clearance. Moreover, compared with lbLDL, sdLDL contains fewer antioxidant vitamins,^40^ making it highly susceptible to oxidation into cytotoxic oxidized LDL.^41^ Oxidized LDL injures endothelial cells, upregulates MCP-1 (monocyte chemoattractant protein-1), and promotes monocyte adhesion and transmigration, initiating inflammatory cascades that lead to foam cell formation.^16^ sdLDL also has lower sialic acid content than lbLDL, increasing its binding to anionic proteoglycans in the vessel wall and enhancing subendothelial retention, which drives lipid accumulation and accelerates plaque progression.^15^ Finally, sdLDL particles activate the fibrinolytic system and induce production of PAI-1 (plasminogen activator inhibitor-1) and thromboxane A_2_, thereby further exacerbating lesion progression.^42^^,^^43^ Taken together, these mechanisms support that sdLDL particles exert stronger atherogenic effects than lbLDL particles. At comparable LDL-C or non-HDL-C levels, a reduced lbLDL-C fraction indicates sdLDL predominance and thus greater atherogenic potential, explaining the association between low lbLDL-C and increased carotid plaque burden in analyses adjusted for these conventional lipid parameters and in discordance analyses. Notably, the sdLDL-C/LDL-C and sdLDL-C/lbLDL-C ratios capture the balance among LDL subfractions with differing atherogenic potential, thereby providing additional atherosclerotic risk information beyond absolute concentrations alone.

Nowadays, statins are widely used in clinical practice to regulate lipid levels, and several studies have shown that they effectively reduce sdLDL-C levels.^39^ However, residual risk of CVD remains even under optimal statin therapy.^44^ This residual risk could be explained by the fact that statins did not reduce the proportion of sdLDL-C because statins lower not only sdLDL-C, but also larger LDL subfractions, leading to disproportionate reductions among LDL-C subtypes.^45^ Thus, the development of more drugs targeting sdLDL-C as a core intervention target will be necessary, with the goal of not only reducing its absolute concentration but also lowering the sdLDL-C/LDL-C fraction and the sdLDL-C/lbLDL-C ratio, thereby shifting LDL particle distribution toward less atherogenic subfractions and potentially mitigating residual cardiovascular risk. In addition to statins, other lipid-lowering drugs also warrant attention. Pemafibrate is a novel selective peroxisome proliferator-activated receptor alpha modulator that can improve the composition of LDL, reducing 20% of sdLDL particles in the cholesterol content and increasing the content of lbLDL particles.^46^ Additionally, the combination therapy of fenofibrate and ezetimibe, compared with statins, regulated sdLDL-C and TG levels, and increased HDL-C levels among patients with type 2 diabetes.^47^ Another clinical trial showed that omega-3 fatty acids could increase LDL particle size and reduce the level of sdLDL-C.^48^ Omega-3 fatty acids also possess significant antioxidant activity, which can protect membrane structure, thereby promoting scavenging of free radicals in sdLDL-C and membrane bilayers.^39^ Finally, niacin also has the potential to decrease sdLDL-C levels, leading to a shift toward massive buoyant LDL particles.^22^ Further randomized controlled trials are needed to investigate the potential cardiovascular benefits of targeting reduction of sdLDL-C and its proportion of total LDL-C.

### Strengths and limitations

Our research comprehensively explored the distinct associations of sdLDL-C, lbLDL-C, sdLDL-C/LDL-C ratio, and sdLDL-C/lbLDL-C ratio with the risk of carotid plaque, and used different methods to perform discordance analyses. We confirmed for the first time that sdLDL-C, sdLDL-C/LDL-C ratio, and sdLDL-C/lbLDL-C ratio, rather than lbLDL-C, were superior to LDL-C and non-HDL-C in identifying individuals at increased risk of carotid plaque. Moreover, both the sdLDL-C/LDL-C ratio and sdLDL-C/lbLDL-C ratio were able to capture additional risk information beyond sdLDL-C. Importantly, another novel finding is that among participants with low LDL-C or non-HDL-C levels, higher lbLDL-C was not associated with increased carotid plaque risk and may even be linked to a reduced risk. These findings may provide novel insights in the development of lipid-lowering therapy and early CVD prevention.

However, this research also had several limitations. First, this study was a cross-section study and it could not assess the causal effects. Future prospective cohort studies are necessary to confirm these associations, and randomized controlled trials are needed to evaluate the effects of interventions aimed at lowering sdLDL-C, the sdLDL-C/LDL-C ratio, and the sdLDL-C/lbLDL-C ratio on reducing residual cardiovascular risk and to establish causality, particularly in individuals with low LDL-C and non-HDL-C levels. Second, the population of this study was a Chinese population. Genetic variations, lifestyle factors, and environmental influences across different ethnic groups may impact lipid profiles and cardiovascular risk, potentially limiting the generalizability of the current findings. Therefore, further studies in diverse populations are needed to validate these results and evaluate the broader applicability of our findings beyond the Chinese population. Finally, although we adjusted for several potential confounders, other unmeasured or residual confounders, such as dietary factors, unrecorded medication use, psychological factors, and genetic factors, may still affect our findings.

## Conclusions

In the general population, sdLDL-C, sdLDL-C/LDL-C ratio, and sdLDL-C/lbLDL-C ratio exhibited stronger associations with carotid plaque risk than did lbLDL-C. Additionally, discordantly high sdLDL-C, sdLDL-C/LDL-C ratio, and sdLDL-C/lbLDL-C ratio relative to LDL-C or non-HDL-C were associated with increased carotid plaque risk, whereas discordantly high lbLDL-C was linked to reduced risk. Notably, the sdLDL-C/LDL-C and sdLDL-C/lbLDL-C ratios could convey additional risk information beyond sdLDL-C. Our findings indicate that sdLDL-C and the ratios of sdLDL-C to LDL-C and to lbLDL-C may serve as novel targets for the prevention and early intervention of carotid plaque and early-stage CVD, even among individuals with optimal LDL-C or non-HDL-C levels.

## Funding Support and Author Disclosures

This work was funded by Capital's Funds for Health Improvement and Research (grant 2024-1G-4033) and the National Natural Science Foundation of China (82073668 to Dr Tao). The funder had no role in the design and conduct of the study; collection, analysis, and interpretation of the data; and preparation, review, or approval of the manuscript. The authors have reported that they have no relationships relevant to the contents of this paper to disclose.
