# Supplementary material for: Discordance of Small Dense LDL Cholesterol Beyond LDL Cholesterol or Non–HDL Cholesterol and Carotid Plaque
Source: JACC Asia. 2025 Jul 8;5(8):1012–28. doi: 10.1016/j.jacasi.2025.04.015 (PMC12426842; doi:10.1016/j.jacasi.2025.04.015)
Supplement: Supplemental Figures 1-8 and Supplemental Tables 1-12 [file mmc1.docx]

**Supplemental Material**

**Supplemental Figure 1.** Study flowchart of participant selection

**Supplemental Figure 2.** The missing proportion and missing pattern of covariates

**Supplemental Figure 3.** Comparison proportion of carotid plaques according to discordance and concordance between sdLDL-C, lb LDL-C and LDL-C or non-HDL-C

**Supplemental Figure 4.** Comparison proportion of carotid plaques according to discordance and concordance of sdLDL-C/LDL-C ratio and sdLDL-C/lbLDL-C ratio with LDL-C, non-HDL-C or sdLDL-C

**Supplemental Figure 5.** Receiver operating characteristic (ROC) analysis in models considering LDL-C and other lipid parameters

**Supplemental Figure 6.** Receiver operating characteristic (ROC) analysis in models considering non-HDL-C and other lipid parameters

**Supplemental Figure 7.** Decision curve analysis (DCA) of models considering LDL-C and other lipid parameters

**Supplemental Figure 8.** Decision curve analysis (DCA) of models considering non-HDL-C and other lipid parameters

**Supplemental Table 1.** Associations between traditional lipid parameters and carotid plaque

**Supplemental Table 2.** Associations of sd LDL-C, lb LDL-C, sd LDL-C/LDL-C, sd LDL-C/lb LDL-C with carotid plaque, after further adjustment for other lipid parameters

**Supplemental Table 3.** Associations between discordant sd LDL-C, lb LDL-C with LDL-C or non-HDL-C and carotid plaque after excluding participants with the usage of antidiabetic medication, antihypertension medication, lipid-lowering medication

**Supplemental Table 4.** Associations between discordant sd LDL-C/LDL-C, sd LDL-C/lb LDL-C with LDL-C, non-HDL-C or sd LDL-C and carotid plaque after excluding participants with the usage of antidiabetic medication, antihypertension medication, lipid-lowering medication

**Supplemental Table 5.** Associations between discordant sd LDL-C, lb LDL-C with LDL-C or non-HDL-C and carotid plaque among the participants without dyslipidemia

**Supplemental Table 6.** Associations between discordant sd LDL-C/LDL-C, sd LDL-C/lb LDL-C with LDL-C, non-HDL-C or sd LDL-C and carotid plaque among the participants without dyslipidemia

**Supplemental Table 7.** Associations between discordant sd LDL-C, lb LDL-C with LDL-C or non-HDL-C and carotid plaque among the participants without cardiovascular diseases

**Supplemental Table 8.** Associations between discordant sd LDL-C/LDL-C, sd LDL-C/lb LDL-C with LDL-C, non-HDL-C or sd LDL-C and carotid plaque among the participants without cardiovascular diseases

**Supplemental Table 9.** Associations between discordant sd LDL-C, lb LDL-C with LDL-C or non-HDL-C and carotid plaque in the participants without missing covariate data

**Supplemental Table 10.** Associations between discordant sd LDL-C/LDL-C, sd LDL-C/lb LDL-C with LDL-C, non-HDL-C or sd LDL-C and carotid plaque in the participants without missing covariate data

**Supplemental Table 11.** Associations between discordant sd LDL-C, lb LDL-C with LDL-C or non-HDL-C and carotid plaque after adjusting for TG and RC

**Supplemental Table 12.** Associations between discordant sd LDL-C/LDL-C, sd LDL-C/lb LDL-C with LDL-C, non-HDL-C or sd LDL-C and carotid plaque after adjusting for TG and RC

22769 participants enrolled in BHMC in 2020 and 2021

39 participants diagnosed with malignant tumor were excluded.

848 participants without color doppler ultrasound examination of carotid artery and diagnostic information of CVD were excluded.

1513 participants without examination of sdLDL-C were excluded.

20369 participants were eligible and enrolled in the final analysis

**Supplemental Figure 1. Study flowchart of participant selection**

Abbreviations: BHMC, Beijing Health Management Cohort; CVD, cardiovascular diseases; sdLDL-C, small dense low-density lipoprotein cholesterol.


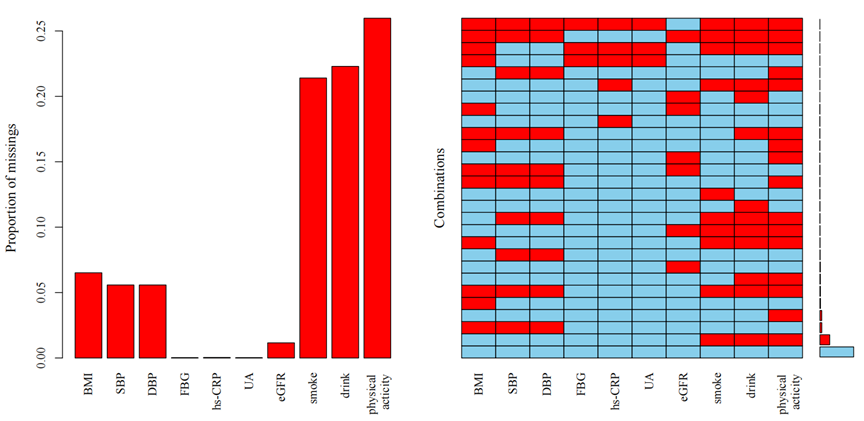


**Supplemental Figure 2. The missing proportion and missing pattern of covariates**

The left panel displays proportion of missing values on each variable, The right panel expresses missing data pattern. Red indicates missing values, while blue indicates no missing values in the right panel.

Abbreviations: BMI, body mass index; SBP, systolic blood pressure; DBP, diastolic blood pressure; FBG, fasting blood glucose; hs-CRP, high-sensitivity C-reactive protein; UA, uric acid; eGFR, estimated glomerular filtration rate.


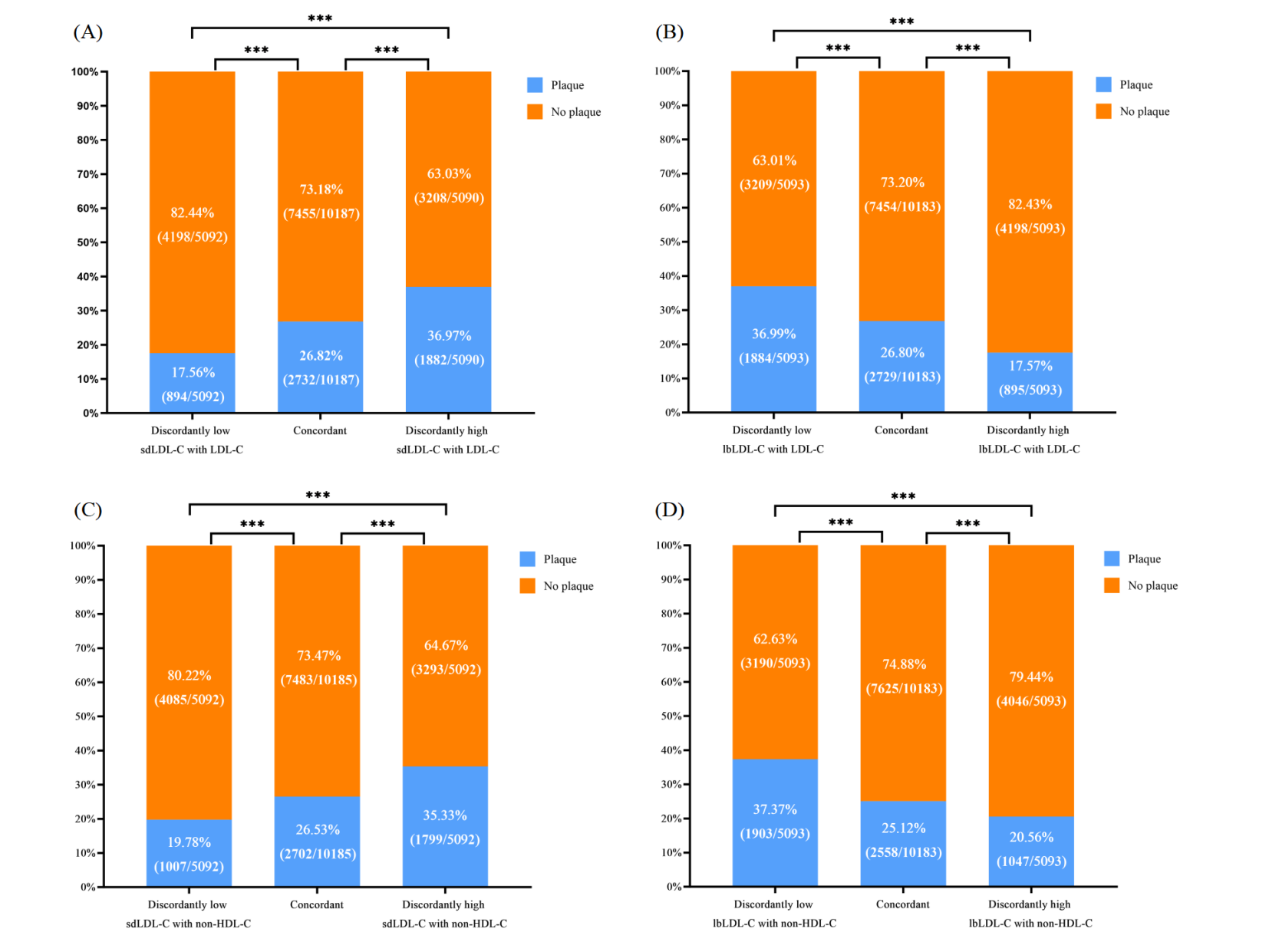


**Supplemental Figure 3. Comparison proportion of carotid plaques** **according to discordance and concordance between sdLDL-C, lbLDL-C and LDL-C or non-HDL-C**

(A) discordant or concordant values of sdLDL-C and LDL-C; (B) discordant or concordant values of lbLDL-C and LDL-C; (C) discordant or concordant values of sdLDL-C and non-HDL-C; (D) discordant or concordant values of lbLDL-C and non-HDL-C.

Discordant low sdLDL-C or lbLDL-C, <25th percentile residual; Concordant, 25th–75th percentile residual; discordant high sdLDL-C or lbLDL-C, >75th percentile residual.

Abbreviations: sdLDL-C, small dense low-density lipoprotein cholesterol; LDL-C, low-density lipoprotein cholesterol; lbLDL-C, large-buoyant low-density lipoprotein cholesterol; non-HDL-C, non-high-density lipoprotein cholesterol.

*** indicates that the Bonferroni correction results show *P* < 0.001, demonstrating that the difference between the two groups is statistically significant.

**
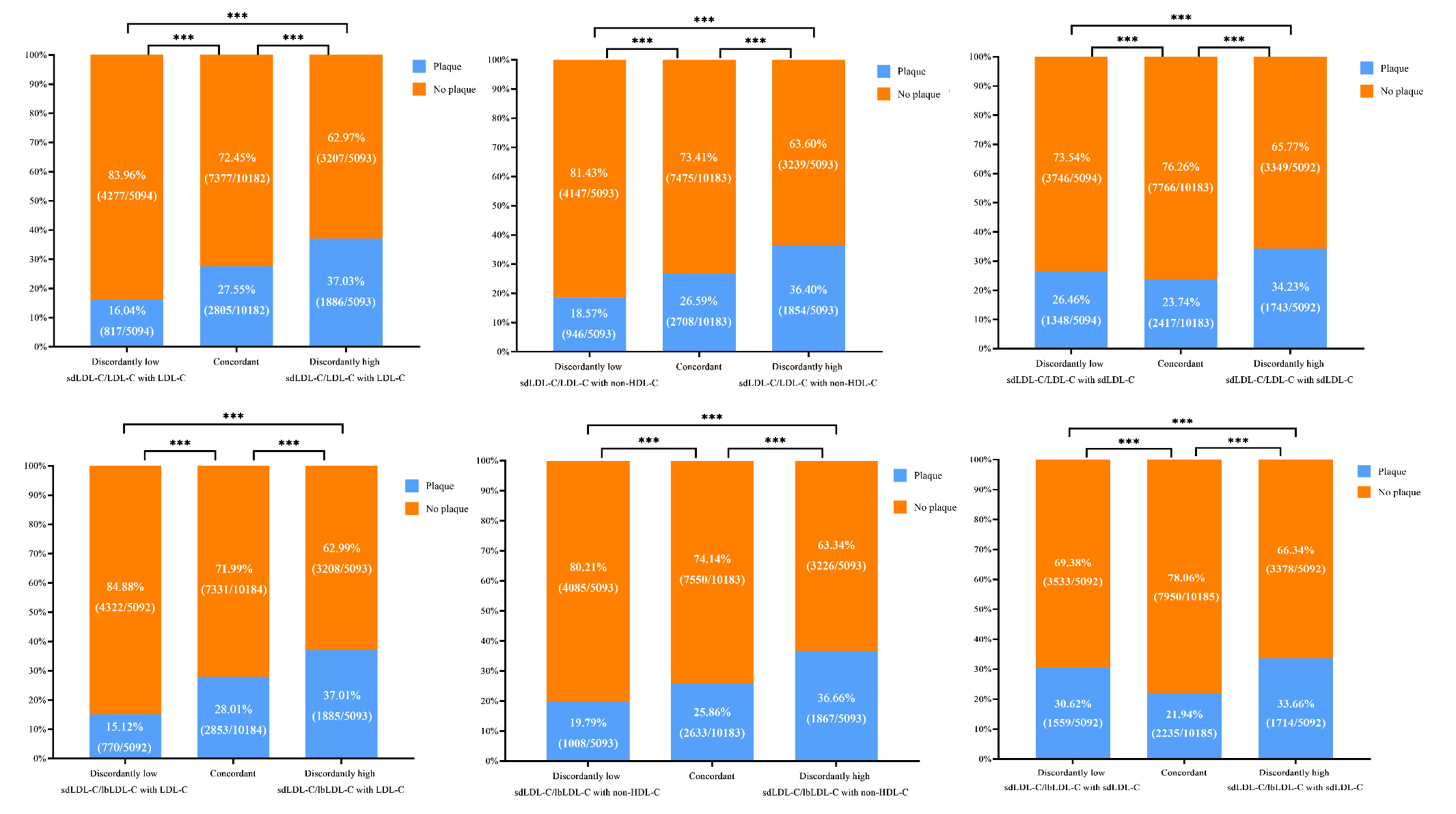
**

**Supplemental Figure 4. Comparison proportion of carotid plaques** **according to discordance and concordance of sdLDL-C/LDL-C ratio and sdLDL-C/lbLDL-C ratio with LDL-C, non-HDL-C or sdLDL-C.**

(A) discordant or concordant values of sdLDL-C/LDL-C and LDL-C; (B) discordant or concordant values of sdLDL-C/LDL-C and non-HDL-C; (C) discordant or concordant values of sdLDL-C/LDL-C and sdLDL-C; (D) discordant or concordant values of sdLDL-C/lbLDL-C and LDL-C; (E) discordant or concordant values of sdLDL-C/lbLDL-C and non-HDL-C; (F) discordant or concordant values of sdLDL-C/lbLDL-C and sdLDL-C.

Discordant low sdLDL-C/LDL-C or sdLDL-C/lbLDL-C, <25th percentile residual; Concordant, 25th–75th percentile residual; discordant high sdLDL-C/LDL-C or sdLDL-C/lbLDL-C, >75th percentile residual.

Abbreviations: sdLDL-C, small dense low-density lipoprotein cholesterol; lbLDL-C, large-buoyant low-density lipoprotein cholesterol; LDL-C, low-density lipoprotein cholesterol; non-HDL-C, non-high-density lipoprotein cholesterol; sd LDL-C/LDL-C, sd LDL-C divided by LDL-C; sd LDL-C/lb LDL-C, sd LDL-C divided by lb LDL-C.

*** indicates that the Bonferroni correction results show *P* < 0.001, demonstrating that the difference between the two groups is statistically significant.


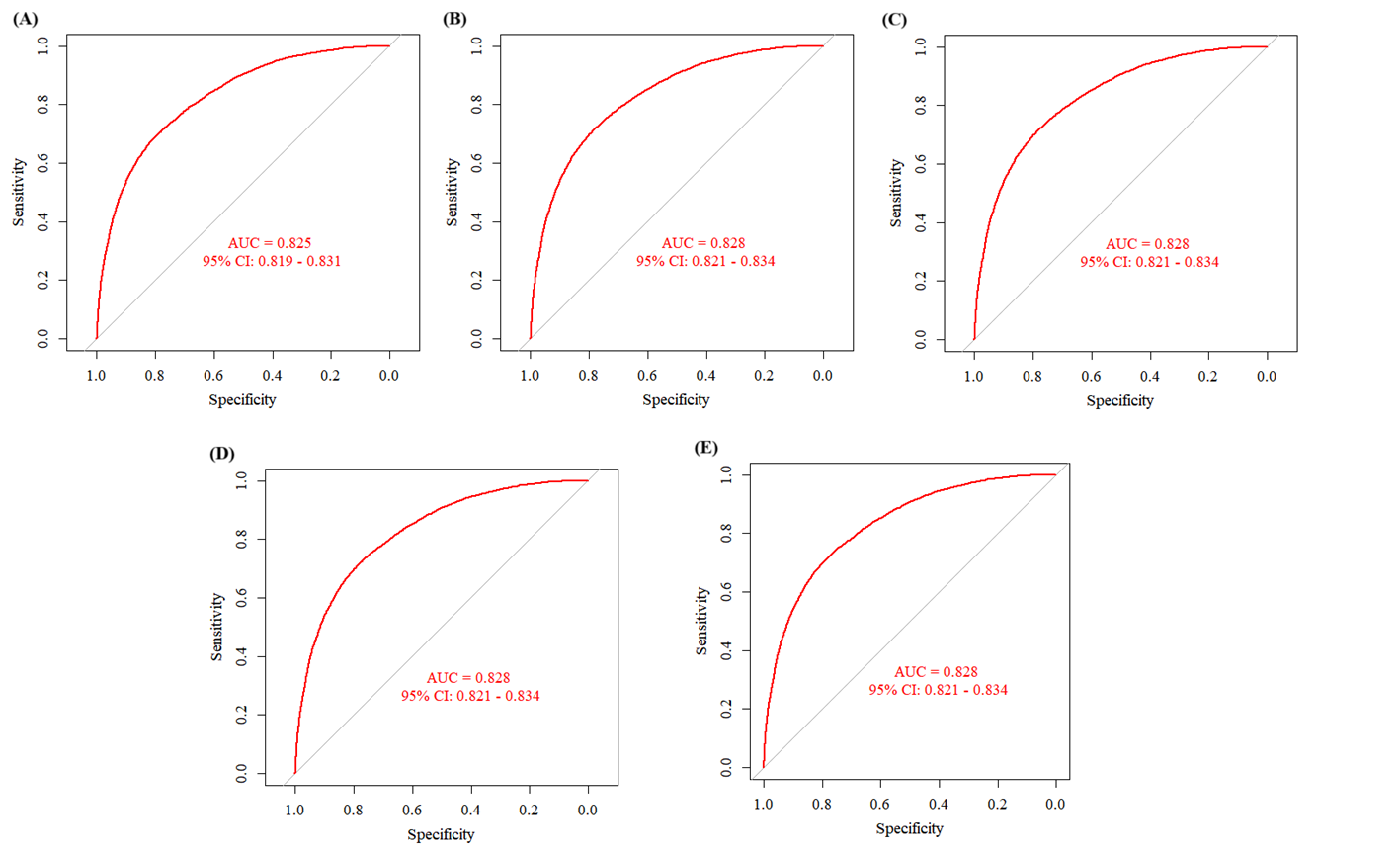


**Supplemental Figure 5. Receiver operating characteristic (ROC) analysis in models considering LDL-C and other lipid parameters**

Models included age, sex, body mass index, diabetes, hypertension, cardiovascular diseases, UA, eGFR, hs-CRP, smoking status, drinking status, use of antidiabetic medication, antihypertensive medication, lipid-lowering medication, physical activity, and various lipid parameters.

1. ROC of the model considering only LDL-C; (B) ROC of the model considering LDL-C and sd LDL-C; (C) ROC of the model considering LDL-C and lb LDL-C; (D) ROC of the model considering LDL-C and sd LDL-C/LDL-C; (E) ROC of the model considering LDL-C and sd LDL-C/lb LDL-C.

Abbreviations: AUC, area under the curve; LDL-C, low-density lipoprotein cholesterol; sd LDL-C, small dense low-density lipoprotein cholesterol; lb LDL-C, large buoyant low-density lipoprotein cholesterol; sdLDL-C/LDL-C, sdLDL-C divided by total LDL-C; sd LDL-C/lb LDL-C, sd LDL-C divided by lb LDL-C; UA, uric acid; eGFR, estimated glomerular filtration rate; hs-CRP, hypersensitivity C-reactive protein; CI, confidence interval.


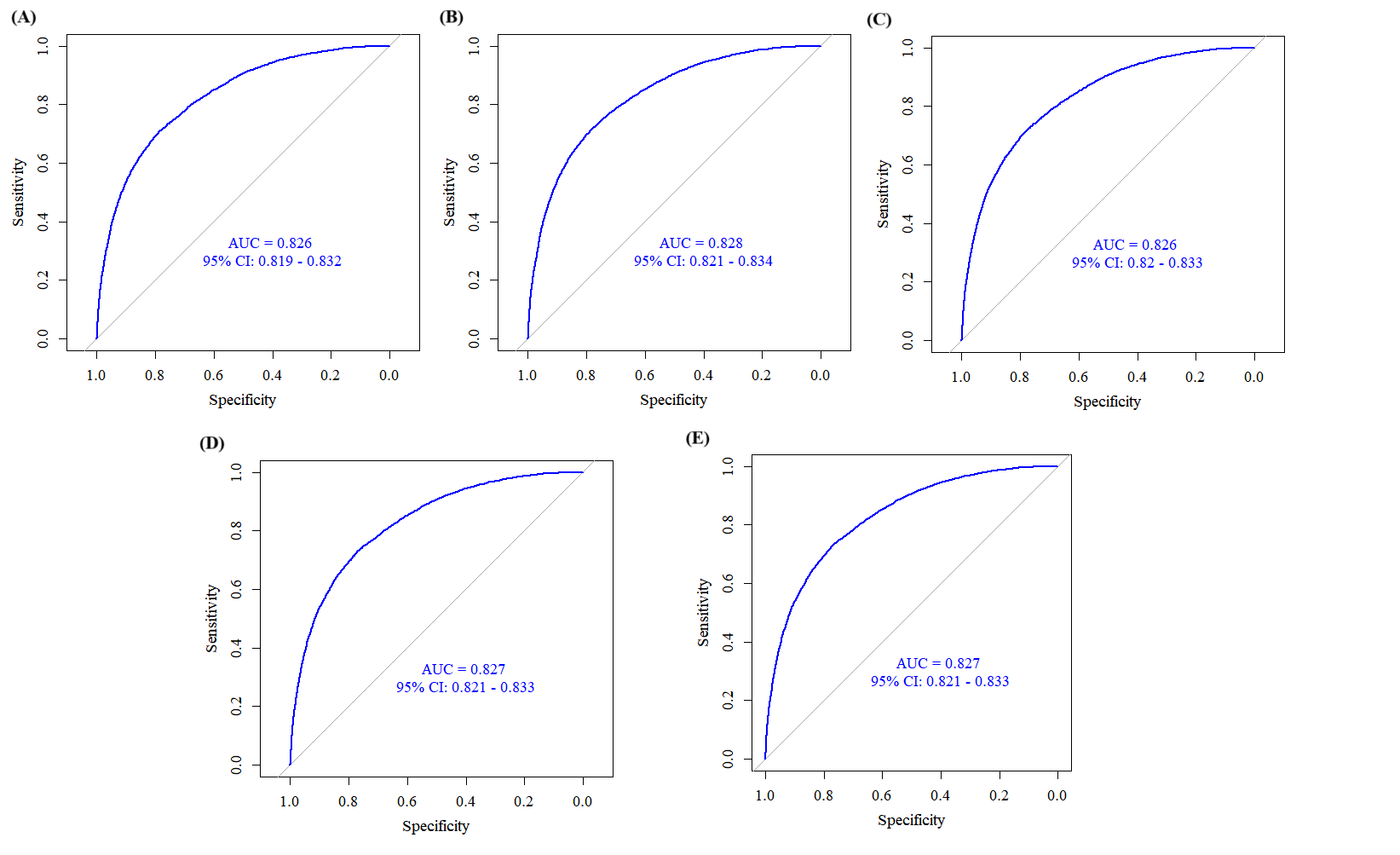


**Supplemental Figure 6. Receiver operating characteristic (ROC) analysis in models considering non-HDL-C and other lipid parameters**

Models included age, sex, body mass index, diabetes, hypertension, cardiovascular diseases, UA, eGFR, hs-CRP, smoking status, drinking status, use of antidiabetic medication, antihypertensive medication, lipid-lowering medication, physical activity, and various lipid parameters.

1. ROC of the model considering only non-HDL-C; (B) ROC of the model considering non-HDL-C and sd LDL-C; (C) ROC of the model considering non-HDL-C and lb LDL-C; (D) ROC of the model considering non-HDL-C and sd LDL-C/LDL-C; (E) ROC of the model considering non-HDL-C and sd LDL-C/lb LDL-C.

Abbreviations: AUC, area under the curve; non-HDL-C, non-high-density lipoprotein cholesterol; sd LDL-C, small dense low-density lipoprotein cholesterol; lb LDL-C, large buoyant low-density lipoprotein cholesterol; sdLDL-C/LDL-C, sdLDL-C divided by total LDL-C; sd LDL-C/lb LDL-C, sd LDL-C divided by lb LDL-C; UA, uric acid; eGFR, estimated glomerular filtration rate; hs-CRP, hypersensitivity C-reactive protein; CI, confidence interval.


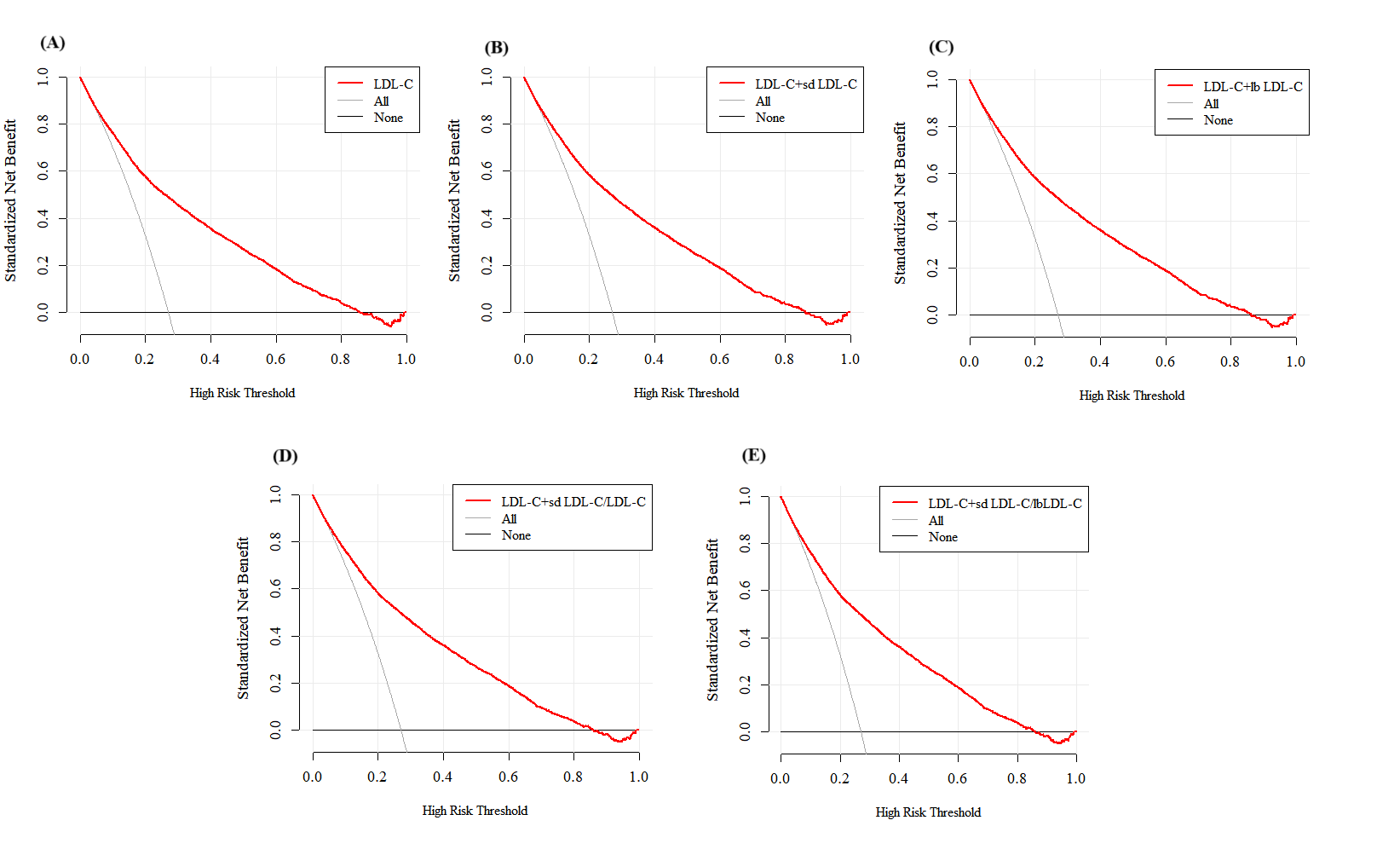


**Supplemental Figure 7.** **Decision curve analysis (DCA) of models considering LDL-C and other lipid parameters**

Models included age, sex, body mass index, diabetes, hypertension, cardiovascular diseases, UA, eGFR, hs-CRP, smoking status, drinking status, use of antidiabetic medication, antihypertensive medication, lipid-lowering medication, physical activity, and various lipid parameters.

1. DCA of the model considering only LDL-C; (B) DCA of the model considering LDL-C and sd LDL-C; (C) DCA of the model considering LDL-C and lb LDL-C; (D) DCA of the model considering LDL-C and sd LDL-C/LDL-C; (E) DCA of the model considering LDL-C and sd LDL-C/lb LDL-C.

A horizontal line indicates that all samples are negative and not treated, with a net benefit of zero. An oblique line indicates that all samples are positive. The net benefit has a negative slope.

Abbreviations: DCA, decision curve analysis; LDL-C, low-density lipoprotein cholesterol; sd LDL-C, small dense low-density lipoprotein cholesterol; lb LDL-C, large buoyant low-density lipoprotein cholesterol; sdLDL-C/LDL-C, sdLDL-C divided by total LDL-C; sd LDL-C/lb LDL-C, sd LDL-C divided by lb LDL-C; UA, uric acid; eGFR, estimated glomerular filtration rate; hs-CRP, hypersensitivity C-reactive protein.


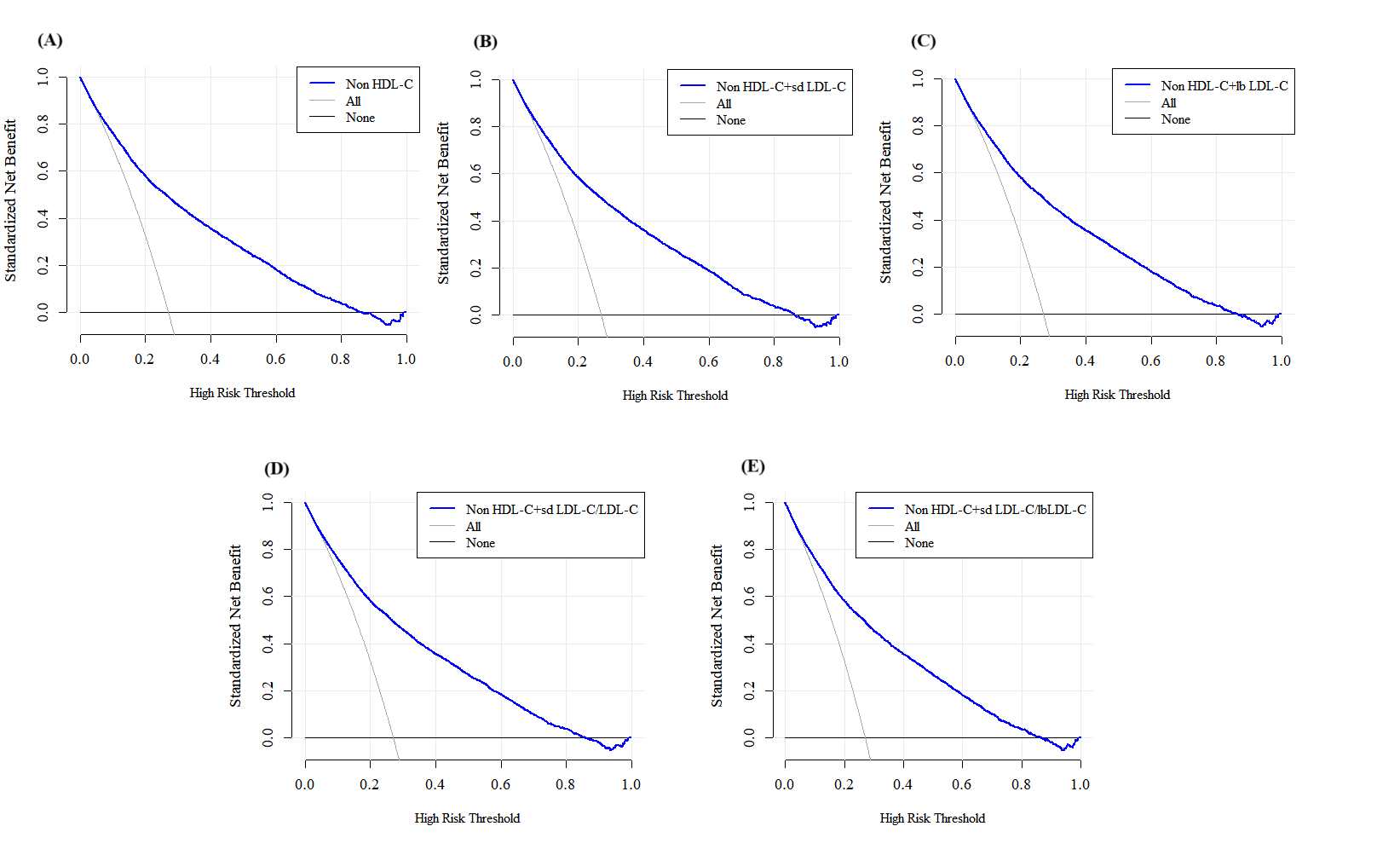


**Supplemental Figure 8. Decision curve analysis (DCA) of models considering non-HDL-C and other lipid parameters**

Models included age, sex, body mass index, diabetes, hypertension, cardiovascular diseases, UA, eGFR, hs-CRP, smoking status, drinking status, use of antidiabetic medication, antihypertensive medication, lipid-lowering medication, physical activity, and various lipid parameters.

1. DCA of the model considering only non-HDL-C; (B) DCA of the model considering non-HDL-C and sd LDL-C; (C) DCA of the model considering non-HDL-C and lb LDL-C; (D) DCA of the model considering non-HDL-C and sd LDL-C/LDL-C; (E) DCA of the model considering non-HDL-C and sd LDL-C/lb LDL-C.

A horizontal line indicates that all samples are negative and not treated, with a net benefit of zero. An oblique line indicates that all samples are positive. The net benefit has a negative slope.

Abbreviations: DCA, decision curve analysis; non-HDL-C, non-high-density lipoprotein cholesterol; sd LDL-C, small dense low-density lipoprotein cholesterol; lb LDL-C, large buoyant low-density lipoprotein cholesterol; sdLDL-C/LDL-C, sdLDL-C divided by total LDL-C; sd LDL-C/lb LDL-C, sd LDL-C divided by lb LDL-C; UA, uric acid; eGFR, estimated glomerular filtration rate; hs-CRP, hypersensitivity C-reactive protein.

**Supplemental Table 1. Associations between traditional lipid parameters and carotid plaque**

|  | **Model 1** | ***P* value** | **Model 2** | ***P* value** |
| --- | --- | --- | --- | --- |
| **LDL-C, mmol/L** |  |  |  |  |
| Categorical |  |  |  |  |
| Quartile 1 | Reference |  | Reference |  |
| Quartile 2 | 0.944 (0.851, 1.048) | 0.280 | 0.997 (0.891, 1.115) | 0.953 |
| Quartile 3 | 1.153 (1.042, 1.276) | 0.006 | 1.214 (1.088, 1.353) | 0.001 |
| Quartile 4 | 1.594 (1.445, 1.759) | <0.001 | 1.617 (1.453, 1.799) | <0.001 |
| Per 1-SD increase | 1.209 (1.168, 1.251) | <0.001 | 1.207 (1.163, 1.253) | <0.001 |
| **Non-HDL-C, mmol/L** |  |  |  |  |
| Categorical |  |  |  |  |
| Quartile 1 | Reference |  | Reference |  |
| Quartile 2 | 1.138 (1.024, 1.265) | 0.017 | 1.141 (1.018, 1.279) | 0.024 |
| Quartile 3 | 1.336 (1.205, 1.482) | <0.001 | 1.310 (1.171, 1.465) | <0.001 |
| Quartile 4 | 1.878 (1.699, 2.077) | <0.001 | 1.746 (1.563, 1.951) | <0.001 |
| Per 1-SD increase | 1.281 (1.237, 1.326) | <0.001 | 1.245 (1.198, 1.293) | <0.001 |
| **HDL-C, mmol/L** |  |  |  |  |
| Categorical |  |  |  |  |
| Quartile 1 | Reference |  | Reference |  |
| Quartile 2 | 0.864 (0.787, 0.949) | 0.002 | 0.893 (0.808, 0.988) | 0.029 |
| Quartile 3 | 0.780 (0.706, 0.861) | <0.001 | 0.819 (0.735, 0.913) | <0.001 |
| Quartile 4 | 0.698 (0.625, 0.778) | <0.001 | 0.720 (0.637, 0.814) | <0.001 |
| Per 1-SD increase | 0.878 (0.843, 0.914) | <0.001 | 0.888 (0.848, 0.929) | <0.001 |
| **TC, mmol/L** |  |  |  |  |
| Categorical |  |  |  |  |
| Quartile 1 | Reference |  | Reference |  |
| Quartile 2 | 1.042 (0.939, 1.156) | 0.439 | 1.094 (0.978, 1.223) | 0.116 |
| Quartile 3 | 1.234 (1.115, 1.366) | <0.001 | 1.217 (1.091, 1.358) | <0.001 |
| Quartile 4 | 1.764 (1.599, 1.946) | <0.001 | 1.657 (1.489, 1.845) | <0.001 |
| Per 1-SD increase | 1.236 (1.195, 1.280) | <0.001 | 1.199 (1.155, 1.244) | <0.001 |
| **TG, mmol/L** |  |  |  |  |
| Categorical |  |  |  |  |
| Quartile 1 | Reference |  | Reference |  |
| Quartile 2 | 1.543 (1.380, 1.726) | <0.001 | 1.388 (1.234, 1.562) | <0.001 |
| Quartile 3 | 1.961 (1.758, 2.187) | <0.001 | 1.623 (1.442, 1.827) | <0.001 |
| Quartile 4 | 2.202 (1.974, 2.456) | <0.001 | 1.747 (1.545, 1.976) | <0.001 |
| Per 1-SD increase | 1.178 (1.140, 1.218) | <0.001 | 1.102 (1.062, 1.142) | <0.001 |
| **RC, mmol/L** |  |  |  |  |
| Categorical |  |  |  |  |
| Quartile 1 | Reference |  | Reference |  |
| Quartile 2 | 1.412 (1.267, 1.574) | <0.001 | 1.274 (1.136, 1.429) | <0.001 |
| Quartile 3 | 1.834 (1.653, 2.036) | <0.001 | 1.548 (1.385, 1.730) | <0.001 |
| Quartile 4 | 2.155 (1.945, 2.387) | <0.001 | 1.622 (1.451, 1.814) | <0.001 |
| Per 1-SD increase | 1.211 (1.172, 1.251) | <0.001 | 1.111 (1.072, 1.150) | <0.001 |

Model 1 adjusted for age, sex.

Model 2 adjusted for age, sex, diabetes, hypertension, cardiovascular diseases, UA, eGFR, high‐sensitivity C‐reactive protein, smoking status, drinking status, body mass index, the usage of antidiabetic medication, antihypertension medication, lipid-lowering medication and physical activity.

Abbreviations: LDL-C, low-density lipoprotein cholesterol; non-HDL-C, non-high-density lipoprotein cholesterol; HDL-C, high-density lipoprotein cholesterol; TC, total cholesterol; TG, triglycerides; RC, remnant cholesterol; SD, standard deviation; UA, uric acid; eGFR, estimated glomerular filtration rate; OR, odds ratio; CI, confidence interval.

**Supplemental Table 2. Associations of sd LDL-C, lb LDL-C, sd LDL-C/LDL-C, sd LDL-C/lb LDL-C with carotid plaque, after further adjustment for other lipid parameters**

| **Models** | **Sd LDL-C** | |  | **Lb LDL-C** | |  | **Sd LDL-C/LDL-C** | |  | **Sd LDL-C/lb LDL-C** | |
| --- | --- | --- | --- | --- | --- | --- | --- | --- | --- | --- | --- |
|  | **OR (95%CI)** | ***P* value** |  | **OR (95%CI)** | ***P* value** |  | **OR (95%CI)** | ***P* value** |  | **OR (95%CI)** | ***P* value** |
| **Model+LDL-C** | 1.346 (1.270, 1.427) | <0.001 |  | 0.621 (0.566, 0.682) | <0.001 |  | 1.237 (1.186, 1.289) | <0.001  <0.001  <0.001  0.001 |  | 1.192 (1.147, 1.239) | <0.001 |
| **Model+Non-HDL-C** | 1.364 (1.274, 1.461) | <0.001 |  | 0.896 (0.849, 0.945) | <0.001 |  | 1.160 (1.112, 1.209) | <0.001  <0.001  <0.001  0.001 |  | 1.124 (1.082, 1.168) | <0.001 |
| **Model+HDL-C** | 1.349 (1.291, 1.409) | <0.001 |  | 1.146 (1.103, 1.190) | <0.001 |  | 1.184 (1.128, 1.244) | <0.001  <0.001  <0.001  0.001 |  | 1.126 (1.079, 1.175) | <0.001 |
| **Model+TC** | 1.364 (1.289, 1.444) | <0.001 |  | 0.909 (0.857, 0.963) | 0.001 |  | 1.199 (1.150, 1.249) | <0.001  <0.001  <0.001  0.001 |  | 1.154 (1.111, 1.199) | <0.001 |
| **Model+TG** | 1.363 (1.302, 1.426) | <0.001 |  | 1.150 (1.107, 1.194) | <0.001 |  | 1.215 (1.147, 1.287) | <0.001 |  | 1.114 (1.056, 1.176) | <0.001 |
| **Model+RC** | 1.366 (1.304, 1.431) | <0.001 |  | 1.121 (1.081, 1.163) | <0.001 |  | 1.175 (1.118, 1.236) | <0.001 |  | 1.104 (1.054, 1.157) | <0.001 |

Model adjusted for age, sex, diabetes, hypertension, cardiovascular diseases, UA, eGFR, high‐sensitivity C‐reactive protein, smoking status, drinking status, body mass index, the usage of antidiabetic medication, antihypertension medication, lipid-lowering medication, physical activity and different lipid parameters.

ORs are shown for each lipid parameter per 1-SD increase.

Abbreviations: SD, standard deviation; LDL-C, low-density lipoprotein cholesterol; non-HDL-C, non-high-density lipoprotein cholesterol; HDL-C, high-density lipoprotein cholesterol; TC, total cholesterol; TG, triglycerides; RC, remnant cholesterol; lb LDL-C, large buoyant low-density lipoprotein cholesterol; sd LDL-C, small dense low-density lipoprotein cholesterol; sd LDL-C/LDL-C, sd LDL-C divided by LDL-C; sd LDL-C/lb LDL-C, sd LDL-C divided by lb LDL-C; UA, uric acid; eGFR, estimated glomerular filtration rate; OR, odds ratio; CI, confidence interval.

**Supplemental Table 3.** **Associations between discordant sd LDL-C, lb LDL-C with LDL-C or non-HDL-C and carotid plaque after excluding participants with** **the usage of antidiabetic medication, antihypertension medication, lipid-lowering medication**

| **Groups** | **OR (95%CI)** | ***P* value** |
| --- | --- | --- |
| **Sd LDL-C and LDL-C** |  |  |
| Discordant low sd LDL-C | 0.840 (0.754, 0.937) | 0.002 |
| Concordant | Reference |  |
| Discordant high sd LDL-C | 1.422 (1.291, 1.567) | <0.001 |
| **Sd LDL-C and non-HDL-C** |  |  |
| Discordant low sd LDL-C | 0.907 (0.815, 1.009) | 0.072 |
| Concordant | Reference |  |
| Discordant high sd LDL-C | 1.401 (1.272, 1.543) | <0.001 |
| **Lb LDL-C and LDL-C** |  |  |
| Discordant low lb LDL-C | 1.424 (1.292, 1.569) | <0.001 |
| Concordant | Reference |  |
| Discordant high lb LDL-C | 0.841 (0.754, 0.938) | 0.002 |
| **Lb LDL-C and non-HDL-C** |  |  |
| Discordant low lb LDL-C | 1.191 (1.082, 1.312) | <0.001 |
| Concordant | Reference |  |
| Discordant high lb LDL-C | 0.950 (0.857, 1.053) | 0.332 |

Models adjusted for age, sex, BMI, diabetes, hypertension, CVD, UA, eGFR, hs-CRP, smoking status, drinking status and physical activity.

Abbreviations: sd LDL-C, small dense low-density lipoprotein cholesterol; LDL-C, low-density lipoprotein cholesterol; non HDL-C, non-high-density lipoprotein cholesterol; lb LDL-C, large buoyant low-density lipoprotein cholesterol; BMI, body mass index; CVD, Cardiovascular disease; UA, uric acid; eGFR, estimated glomerular filtration rate; hs-CRP, high-sensitivity C-reactive protein; OR, odds ratio; CI, confidence interval.

**Supplemental Table 4. Associations between discordant sdLDL-C/LDL-C, sd LDL-C/lb LDL-C with LDL-C, non-HDL-C or sdLDL-C and carotid plaque after excluding participants with the usage of antidiabetic medication, antihypertension medication, lipid-lowering medication**

| **Groups** | **OR (95%CI)** | ***P* value** |
| --- | --- | --- |
| **Sd LDL-C/LDL-C and LDL-C** |  |  |
| Discordant low sd LDL-C/LDL-C | 0.777 (0.694, 0.870) | <0.001 |
| Concordant | Reference |  |
| Discordant high sd LDL-C/LDL-C | 1.347 (1.224, 1.483) | <0.001 |
| **Sd LDL-C/LDL-C and non-HDL-C** |  |  |
| Discordant low sd LDL-C/LDL-C | 0.873 (0.784, 0.972) | 0.013 |
| Concordant | Reference |  |
| Discordant high sd LDL-C/LDL-C | 1.269 (1.152, 1.396) | <0.001 |
| **Sd LDL-C/LDL-C and sd LDL-C** |  |  |
| Discordant low sd LDL-C/LDL-C | 0.796 (0.707, 0.897) | <0.001 |
| Concordant | Reference |  |
| Discordant high sd LDL-C/LDL-C | 1.432 (1.284, 1.597) | <0.001 |
| **Sd LDL-C/lb LDL-C and LDL-C** |  |  |
| Discordant low sd LDL-C/lb LDL-C | 0.723 (0.644, 0.812) | <0.001 |
| Concordant | Reference |  |
| Discordant high sd LDL-C/lb LDL-C | 1.414 (1.285, 1.555) | <0.001 |
| **Sd LDL-C/lb LDL-C and non-HDL-C** |  |  |
| Discordant low sd LDL-C/lb LDL-C | 0.885 (0.796, 0.984) | 0.024 |
| Concordant | Reference |  |
| Discordant high sd LDL-C/lb LDL-C | 1.291 (1.172, 1.421) | <0.001 |
| **Sd LDL-C/lb LDL-C and sd LDL-C** |  |  |
| Discordant low sd LDL-C/lb LDL-C | 0.871 (0.767, 0.988) | 0.032 |
| Concordant | Reference |  |
| Discordant high sd LDL-C/lb LDL-C | 1.447 (1.296, 1.615) | <0.001 |

Models adjusted for age, sex, BMI, diabetes, hypertension, CVD, UA, eGFR, hs-CRP, smoking status, drinking status and physical activity.

Abbreviations: sd LDL-C, small dense low-density lipoprotein cholesterol; LDL-C, low-density lipoprotein cholesterol; non HDL-C, non-high-density lipoprotein cholesterol; lb LDL-C, large buoyant low-density lipoprotein cholesterol; sdLDL-C/LDL-C, sdLDL-C divided by total LDL-C; sd LDL-C/lb LDL-C, sd LDL-C divided by lb LDL-C; BMI, body mass index; CVD, Cardiovascular disease; UA, uric acid; eGFR, estimated glomerular filtration rate; hs-CRP, high-sensitivity C-reactive protein; OR, odds ratio; CI, confidence interval.

**Supplemental Table 5. Associations between discordant sdLDL-C, lb LDL-C with LDL-C or non-HDL-C and carotid plaque among the participants without dyslipidemia**

| **Groups** | **OR (95%CI)** | ***P* value** |
| --- | --- | --- |
| **Sd LDL-C and LDL-C** |  |  |
| Discordant low sd LDL-C | 0.883 (0.776, 1.004) | 0.058 |
| Concordant | Reference |  |
| Discordant high sd LDL-C | 1.335 (1.191, 1.496) | <0.001 |
| **Sd LDL-C and non-HDL-C** |  |  |
| Discordant low sd LDL-C | 0.977 (0.860, 1.109) | 0.716 |
| Concordant | Reference |  |
| Discordant high sd LDL-C | 1.399 (1.248, 1.568) | <0.001 |
| **Lb LDL-C and LDL-C** |  |  |
| Discordant low lb LDL-C | 1.339 (1.195, 1.500) | <0.001 |
| Concordant | Reference |  |
| Discordant high lb LDL-C | 0.879 (0.773, 1.000) | 0.049 |
| **Lb LDL-C and non-HDL-C** |  |  |
| Discordant low lb LDL-C | 1.341 (1.198, 1.501) | <0.001 |
| Concordant | Reference |  |
| Discordant high lb LDL-C | 0.791 (0.694, 0.902) | <0.001 |

Models adjusted for age, sex, BMI, diabetes, hypertension, CVD, UA, eGFR, hs-CRP, smoking status, drinking status, the usage of antidiabetic medication, antihypertension medication, and physical activity.

Abbreviations: sd LDL-C, small dense low-density lipoprotein cholesterol; LDL-C, low-density lipoprotein cholesterol; non HDL-C, non-high-density lipoprotein cholesterol; lb LDL-C, large buoyant low-density lipoprotein cholesterol; BMI, body mass index; CVD, cardiovascular disease; UA, uric acid; eGFR, estimated glomerular filtration rate; hs-CRP, high-sensitivity C-reactive protein; OR, odds ratio; CI, confidence interval.

**Supplemental Table 6. Associations between discordant sdLDL-C/LDL-C,** **sd LDL-C/lb LDL-C with LDL-C, non-HDL-C or sdLDL-C and carotid plaque among the participants without dyslipidemia**

| **Groups** | **OR (95%CI)** | ***P* value** |
| --- | --- | --- |
| **Sd LDL-C/LDL-C and LDL-C** |  |  |
| Discordant low sd LDL-C/LDL-C | 0.790 (0.692, 0.902) | 0.001 |
| Concordant | Reference |  |
| Discordant high sd LDL-C/LDL-C | 1.275 (1.140, 1.427) | <0.001 |
| **Sd LDL-C/LDL-C and non-HDL-C** |  |  |
| Discordant low sd LDL-C/LDL-C | 0.847 (0.743, 0.965) | 0.012 |
| Concordant | Reference |  |
| Discordant high sd LDL-C/LDL-C | 1.275 (1.139, 1.428) | <0.001 |
| **Sd LDL-C/LDL-C and sd LDL-C** |  |  |
| Discordant low sd LDL-C/LDL-C | 0.837 (0.734, 0.955) | 0.008 |
| Concordant | Reference |  |
| Discordant high sd LDL-C/LDL-C | 1.394 (1.220, 1.592) | <0.001 |
| **Sd LDL-C/lb LDL-C and LDL-C** |  |  |
| Discordant low sd LDL-C/lb LDL-C | 0.811 (0.711, 0.925) | 0.002 |
| Concordant | Reference |  |
| Discordant high sd LDL-C/lb LDL-C | 1.304 (1.164, 1.462) | <0.001 |
| **Sd LDL-C/lb LDL-C and non-HDL-C** |  |  |
| Discordant low sd LDL-C/lb LDL-C | 0.844 (0.741, 0.960) | 0.010 |
| Concordant | Reference |  |
| Discordant high sd LDL-C/lb LDL-C | 1.262 (1.127, 1.413) | <0.001 |
| **Sd LDL-C/lb LDL-C and sd LDL-C** |  |  |
| Discordant low sd LDL-C/lb LDL-C | 0.875 (0.765, 1.001) | 0.052 |
| Concordant | Reference |  |
| Discordant high sd LDL-C/lb LDL-C | 1.402 (1.232, 1.594) | <0.001 |

Models adjusted for age, sex, BMI, diabetes, hypertension, CVD, UA, eGFR, hs-CRP, smoking status, drinking status, the usage of antidiabetic medication, antihypertension medication and physical activity.

Abbreviations: sd LDL-C, small dense low-density lipoprotein cholesterol; LDL-C, low-density lipoprotein cholesterol; non HDL-C, non-high-density lipoprotein cholesterol; lb LDL-C, large buoyant low-density lipoprotein cholesterol; sdLDL-C/LDL-C, sdLDL-C divided by total LDL-C; sd LDL-C/lb LDL-C, sd LDL-C divided by lb LDL-C; CVD, cardiovascular disease; BMI, body mass index; UA, uric acid; eGFR, estimated glomerular filtration rate; hs-CRP, high-sensitivity C-reactive protein; OR, odds ratio; CI, confidence interval.

**Supplemental Table 7. Associations between discordant sdLDL-C, lb LDL-C with LDL-C or non-HDL-C and carotid plaque among the participants without cardiovascular diseases**

| **Groups** | **OR (95%CI)** | ***P* value** |
| --- | --- | --- |
| **Sd LDL-C and LDL-C** |  |  |
| Discordant low sd LDL-C | 0.809 (0.726, 0.901) | <0.001 |
| Concordant | Reference |  |
| Discordant high sd LDL-C | 1.416 (1.289, 1.555) | <0.001 |
| **Sd LDL-C and non-HDL-C** |  |  |
| Discordant low sd LDL-C | 0.878 (0.790, 0.975) | 0.015 |
| Concordant | Reference |  |
| Discordant high sd LDL-C | 1.393 (1.268, 1.530) | <0.001 |
| **Lb LDL-C and LDL-C** |  |  |
| Discordant low lb LDL-C | 1.411 (1.285, 1.550) | <0.001 |
| Concordant | Reference |  |
| Discordant high lb LDL-C | 0.801 (0.719, 0.892) | <0.001 |
| **Lb LDL-C and non-HDL-C** |  |  |
| Discordant low lb LDL-C | 1.180 (1.074, 1.295) | 0.001 |
| Concordant | Reference |  |
| Discordant high lb LDL-C | 0.892 (0.806, 0.987) | 0.027 |

Models adjusted for age, sex, BMI, diabetes, hypertension, UA, eGFR, hs-CRP, smoking status, drinking status, the usage of antidiabetic medication, antihypertension medication and lipid-lowering medication and physical activity.

Abbreviations: sd LDL-C, small dense low-density lipoprotein cholesterol; LDL-C, low-density lipoprotein cholesterol; non HDL-C, non-high-density lipoprotein cholesterol; lb LDL-C, large buoyant low-density lipoprotein cholesterol; BMI, body mass index; UA, uric acid; eGFR, estimated glomerular filtration rate; hs-CRP, high-sensitivity C-reactive protein; OR, odds ratio; CI, confidence interval.

**Supplemental Table 8. Associations between discordant sdLDL-C/LDL-C, sd LDL-C/lb LDL-C with LDL-C, non-HDL-C or sdLDL-C and carotid plaque among the participants without cardiovascular diseases**

| **Groups** | **OR (95%CI)** | ***P* value** |
| --- | --- | --- |
| **Sd LDL-C/LDL-C and LDL-C** |  |  |
| Discordant low sd LDL-C/LDL-C | 0.768 (0.688, 0.858) | <0.001 |
| Concordant | Reference |  |
| Discordant high sd LDL-C/LDL-C | 1.312 (1.196, 1.441) | <0.001 |
| **Sd LDL-C/LDL-C and non-HDL-C** |  |  |
| Discordant low sd LDL-C/LDL-C | 0.828 (0.745, 0.921) | <0.001 |
| Concordant | Reference |  |
| Discordant high sd LDL-C/LDL-C | 1.225 (1.116, 1.345) | <0.001 |
| **Sd LDL-C/LDL-C and sd LDL-C** |  |  |
| Discordant low sd LDL-C/LDL-C | 0.824 (0.734, 0.924) | 0.001 |
| Concordant | Reference |  |
| Discordant high sd LDL-C/LDL-C | 1.426 (1.282, 1.586) | <0.001 |
| **Sd LDL-C/lb LDL-C and LDL-C** |  |  |
| Discordant low sd LDL-C/lb LDL-C | 0.726 (0.649, 0.813) | <0.001 |
| Concordant | Reference |  |
| Discordant high sd LDL-C/lb LDL-C | 1.380 (1.257, 1.514) | <0.001 |
| **Sd LDL-C/lb LDL-C and non-HDL-C** |  |  |
| Discordant low sd LDL-C/lb LDL-C | 0.869 (0.783, 0.964) | 0.008 |
| Concordant | Reference |  |
| Discordant high sd LDL-C/lb LDL-C | 1.245 (1.133, 1.368) | <0.001 |
| **Sd LDL-C/lb LDL-C and sd LDL-C** |  |  |
| Discordant low sd LDL-C/lb LDL-C | 0.915 (0.809, 1.035) | 0.156 |
| Concordant | Reference |  |
| Discordant high sd LDL-C/lb LDL-C | 1.474 (1.324, 1.642) | <0.001 |

Models adjusted for age, sex, BMI, diabetes, hypertension, UA, eGFR, hs-CRP, smoking status, drinking status, the usage of antidiabetic medication, antihypertension medication and lipid-lowering medication and physical activity.

Abbreviations: sd LDL-C, small dense low-density lipoprotein cholesterol; LDL-C, low-density lipoprotein cholesterol; non HDL-C, non-high-density lipoprotein cholesterol; lb LDL-C, large buoyant low-density lipoprotein cholesterol; sdLDL-C/LDL-C, sdLDL-C divided by total LDL-C; sd LDL-C/lb LDL-C, sd LDL-C divided by lb LDL-C; BMI, body mass index; UA, uric acid; eGFR, estimated glomerular filtration rate; hs-CRP, high-sensitivity C-reactive protein; OR, odds ratio; CI, confidence interval.

**Supplemental Table 9. Associations between discordant sd LDL-C, lb LDL-C with LDL-C or non-HDL-C and carotid plaque in the participants without missing covariate data**

| **Groups** | **OR (95%CI)** | ***P* value** |
| --- | --- | --- |
| **Sd LDL-C and LDL-C** |  |  |
| Discordant low sd LDL-C | 0.861 (0.759, 0.976) | 0.020 |
| Concordant | Reference |  |
| Discordant high sd LDL-C | 1.439 (1.288, 1.606) | <0.001 |
| **Sd LDL-C and non-HDL-C** |  |  |
| Discordant low sd LDL-C | 0.869 (0.768, 0.983) | 0.026 |
| Concordant | Reference |  |
| Discordant high sd LDL-C | 1.345 (1.205, 1.502) | <0.001 |
| **Lb LDL-C and LDL-C** |  |  |
| Discordant low lb LDL-C | 1.439 (1.288 1.606) | <0.001 |
| Concordant | Reference |  |
| Discordant high lb LDL-C | 0.861 (0.759, 0.976) | 0.020 |
| **Lb LDL-C and non-HDL-C** |  |  |
| Discordant low lb LDL-C | 1.210 (1.083, 1.351) | 0.001 |
| Concordant | Reference |  |
| Discordant high lb LDL-C | 0.992 (0.882, 1.117) | 0.896 |

Models adjusted for age, sex, BMI, diabetes, hypertension, CVD, UA, eGFR, hs-CRP, the usage of antidiabetic medication, antihypertension medication and lipid-lowering medication, smoking status, drinking status and physical activity.

Abbreviations: sd LDL-C, small dense low-density lipoprotein cholesterol; LDL-C, low-density lipoprotein cholesterol; non-HDL-C, non-high-density lipoprotein cholesterol; lb LDL-C, large buoyant low-density lipoprotein cholesterol; BMI, body mass index; CVD, Cardiovascular disease; UA, uric acid; eGFR, estimated glomerular filtration rate; hs-CRP, high-sensitivity C-reactive protein; OR, odds ratio; CI, confidence interval.

**Supplemental Table 10. Associations between discordant sdLDL-C/LDL-C****, sd LDL-C/lb LDL-C with LDL-C, non-HDL-C or sdLDL-C and carotid plaque in the participants without missing covariate data**

| **Groups** | **OR (95%CI)** | ***P* value** |
| --- | --- | --- |
| **Sd LDL-C/LDL-C and LDL-C** |  |  |
| Discordant low sd LDL-C/LDL-C | 0.770 (0.676, 0.877) | <0.001 |
| Concordant | Reference |  |
| Discordant high sd LDL-C/LDL-C | 1.300 (1.165, 1.450) | <0.001 |
| **Sd LDL-C/LDL-C and non-HDL-C** |  |  |
| Discordant low sd LDL-C/LDL-C | 0.856 (0.756, 0.969) | 0.014 |
| Concordant | Reference |  |
| Discordant high sd LDL-C/LDL-C | 1.211 (1.085, 1.351) | 0.001 |
| **Sd LDL-C/LDL-C and sd LDL-C** |  |  |
| Discordant low sd LDL-C/LDL-C | 0.854 (0.746, 0.979) | 0.023 |
| Concordant | Reference |  |
| Discordant high sd LDL-C/LDL-C | 1.504 (1.327, 1.706) | <0.001 |
| **Sd LDL-C/lb LDL-C and LDL-C** |  |  |
| Discordant low sd LDL-C/lb LDL-C | 0.725 (0.635, 0.828) | <0.001 |
| Concordant | Reference |  |
| Discordant high sd LDL-C/lb LDL-C | 1.358 (1.218, 1.515) | <0.001 |
| **Sd LDL-C/lb LDL-C and non-HDL-C** |  |  |
| Discordant low sd LDL-C/lb LDL-C | 0.913 (0.808, 1.032) | 0.145 |
| Concordant | Reference |  |
| Discordant high sd LDL-C/lb LDL-C | 1.239 (1.109, 1.383) | <0.001 |
| **Sd LDL-C/lb LDL-C and sd LDL-C** |  |  |
| Discordant low sd LDL-C/lb LDL-C | 0.947 (0.819, 1.094) | 0.459 |
| Concordant | Reference |  |
| Discordant high sd LDL-C/lb LDL-C | 1.490 (1.312, 1.692) | <0.001 |

Models adjusted for age, sex, BMI, diabetes, hypertension, CVD, UA, eGFR, hs-CRP, the usage of antidiabetic medication, antihypertension medication and lipid-lowering medication, smoking status, drinking status and physical activity.

Abbreviations: sd LDL-C, small dense low-density lipoprotein cholesterol; LDL-C, low-density lipoprotein cholesterol; non HDL-C, non-high-density lipoprotein cholesterol; lb LDL-C, large buoyant low-density lipoprotein cholesterol; sdLDL-C/LDL-C, sdLDL-C divided by total LDL-C; sd LDL-C/lb LDL-C, sd LDL-C divided by lb LDL-C; BMI, body mass index; CVD, Cardiovascular disease; UA, uric acid; eGFR, estimated glomerular filtration rate; hs-CRP, high-sensitivity C-reactive protein; OR, odds ratio; CI, confidence interval.

**Supplemental Table 11. Associations between discordant sd LDL-C, lb LDL-C with LDL-C or non-HDL-C and carotid plaque** **after adjusting for TG and RC**

| **Groups** | **Model 1 (+TG)** | |  | **Model 2 (+RC)** | |
| --- | --- | --- | --- | --- | --- |
|  | **OR (95%CI)** | ***P* value** |  | **OR (95%CI)** | ***P* value** |
| **Sd LDL-C and LDL-C** |  |  |  |  |  |
| Discordant low sd LDL-C | 0.827 (0.747, 0.916) | <0.001 |  | 0.825 (0.746, 0.914) | <0.001 |
| Concordant | Reference |  |  | Reference |  |
| Discordant high sd LDL-C | 1.369 (1.239, 1.512) | <0.001 |  | 1.336 (1.211, 1.473) | <0.001 |
| **Sd LDL-C and non-HDL-C** |  |  |  |  |  |
| Discordant low sd LDL-C | 0.867 (0.785, 0.958) | 0.005 |  | 0.843 (0.763, 0.931) | 0.001 |
| Concordant | Reference |  |  | Reference |  |
| Discordant high sd LDL-C | 1.307 (1.192, 1.432) | <0.001 |  | 1.317 (1.204, 1.441) | <0.001 |
| **Lb LDL-C and LDL-C** |  |  |  |  |  |
| Discordant low lb LDL-C | 1.372 (1.242, 1.517) | <0.001 |  | 1.339 (1.215, 1.477) | <0.001 |
| Concordant | Reference |  |  | Reference |  |
| Discordant high lb LDL-C | 0.829 (0.749, 0.918) | <0.001 |  | 0.828 (0.748, 0.916) | <0.001 |
| **Lb LDL-C and non-HDL-C** |  |  |  |  |  |
| Discordant low lb LDL-C | 1.081 (0.977, 1.197)  210 (1.083, 1.351) | 0.131 |  | 1.067 (0.966, 1.180) | 0.201 |
| Concordant | Reference |  |  | Reference |  |
| Discordant high lb LDL-C | 0.942 (0.856, 1.037) | 0.221 |  | 0.935 (0.849, 1.028) | 0.166 |

Model 1 adjusted for age, sex, BMI, diabetes, hypertension, CVD, UA, eGFR, hs-CRP, use of antidiabetic, antihypertensive, and lipid-lowering medications, smoking status, drinking status, physical activity, and TG.

Model 2 adjusted for age, sex, BMI, diabetes, hypertension, CVD, UA, eGFR, hs-CRP, use of antidiabetic, antihypertensive, and lipid-lowering medications, smoking status, drinking status, physical activity, and RC.

Abbreviations: sd LDL-C, small dense low-density lipoprotein cholesterol; LDL-C, low-density lipoprotein cholesterol; HDL-C, high-density lipoprotein cholesterol; non-HDL-C, non-high-density lipoprotein cholesterol; lb LDL-C, large buoyant low-density lipoprotein cholesterol; RC, remnant cholesterol; TG, triglycerides; BMI, body mass index; CVD, Cardiovascular disease; UA, uric acid; eGFR, estimated glomerular filtration rate; hs-CRP, high-sensitivity C-reactive protein; OR, odds ratio; CI, confidence interval.

**Supplemental Table 12. Associations between discordant sd LDL-C/LDL-C, sd LDL-C/lb LDL-C with LDL-C, non-HDL-C or sd LDL-C and carotid plaque after adjusting for TG and RC**

| **Groups** | **Model 1 (+TG)** | |  | **Model 2 (+RC)** | |
| --- | --- | --- | --- | --- | --- |
|  | **OR (95%CI)** | ***P* value** |  | **OR (95%CI)** | ***P* value** |
| **Sd LDL-C/LDL-C and LDL-C** |  |  |  |  |  |
| Discordant low sd LDL-C/LDL-C | 0.771 (0.694, 0.857) | <0.001 |  | 0.770 (0.694, 0.855) | <0.001 |
| Concordant | Reference |  |  | Reference |  |
| Discordant high sd LDL-C/LDL-C | 1.262 (1.143, 1.393) | <0.001 |  | 1.238 (1.125, 1.363) | <0.001 |
| **Sd LDL-C/LDL-C and non-HDL-C** |  |  |  |  |  |
| Discordant low sd LDL-C/LDL-C | 0.853 (0.771, 0.943) | 0.002 |  | 0.842 (0.761, 0.930) | 0.001 |
| Concordant | Reference |  |  | Reference |  |
| Discordant high sd LDL-C/LDL-C | 1.160 (1.053, 1.277) | 0.003 |  | 1.158 (1.055, 1.270) | 0.002 |
| **Sd LDL-C/LDL-C and sd LDL-C** |  |  |  |  |  |
| Discordant low sd LDL-C/LDL-C | 0.834 (0.747, 0.931) | 0.001 |  | 0.826 (0.740, 0.921) | 0.001 |
| Concordant | Reference |  |  | Reference |  |
| Discordant high sd LDL-C/LDL-C | 1.388 (1.247, 1.546) | <0.001 |  | 1.413 (1.271, 1.570) | <0.001 |
| **Sd LDL-C/lb LDL-C and LDL-C** |  |  |  |  |  |
| Discordant low sd LDL-C/lb LDL-C | 0.714 (0.642, 0.795) | <0.001 |  | 0.715 (0.643, 0.796) | <0.001 |
| Concordant | Reference |  |  | Reference |  |
| Discordant high sd LDL-C/lb LDL-C | 1.306 (1.183, 1.441) | <0.001 |  | 1.276 (1.159, 1.405) | <0.001 |
| **Sd LDL-C/lb LDL-C and non-HDL-C** |  |  |  |  |  |
| Discordant low sd LDL-C/lb LDL-C | 0.880 (0.797, 0.971) | 0.011 |  | 0.865 (0.784, 0.955) | 0.004 |
| Concordant | Reference |  |  | Reference |  |
| Discordant high sd LDL-C/lb LDL-C | 1.188 (1.078, 1.308) | <0.001 |  | 1.183 (1.078, 1.299) | <0.001 |
| **Sd LDL-C/lb LDL-C and sd LDL-C** |  |  |  |  |  |
| Discordant low sd LDL-C/lb LDL-C | 0.932 (0.829, 1.049) | 0.244 |  | 0.925 (0.822, 1.040) | 0.190 |
| Concordant | Reference |  |  | Reference |  |
| Discordant high sd LDL-C/lb LDL-C | 1.364 (1.223, 1.521) | <0.001 |  | 1.393 (1.251, 1.550) | <0.001 |

Model 1 adjusted for age, sex, BMI, diabetes, hypertension, CVD, UA, eGFR, hs-CRP, use of antidiabetic, antihypertensive, and lipid-lowering medications, smoking status, drinking status, physical activity, and TG.

Model 2 adjusted for age, sex, BMI, diabetes, hypertension, CVD, UA, eGFR, hs-CRP, use of antidiabetic, antihypertensive, and lipid-lowering medications, smoking status, drinking status, physical activity, and RC.

Abbreviations: sd LDL-C, small dense low-density lipoprotein cholesterol; LDL-C, low-density lipoprotein cholesterol; HDL-C, high-density lipoprotein cholesterol; non-HDL-C, non-high-density lipoprotein cholesterol; lb LDL-C, large buoyant low-density lipoprotein cholesterol; sd LDL-C/LDL-C, sd LDL-C divided by total LDL-C; sd LDL-C/lb LDL-C, sd LDL-C divided by lb LDL-C; RC, remnant cholesterol; TG, triglycerides; BMI, body mass index; CVD, Cardiovascular disease; UA, uric acid; eGFR, estimated glomerular filtration rate; hs-CRP, high-sensitivity C-reactive protein; OR, odds ratio; CI, confidence interval.
